# Intracellular zinc flux causes reactive oxygen species mediated mitochondrial dysfunction leading to cell death in *Leishmania donovani*

**DOI:** 10.1371/journal.pone.0178800

**Published:** 2017-06-06

**Authors:** Anjali Kumari, Krishn Pratap Singh, Abhishek Mandal, Ranjeet Kumar Paswan, Preeti Sinha, Pradeep Das, Vahab Ali, Sanjiva Bimal, Chandra Shekhar Lal

**Affiliations:** 1 Division of Biochemistry, Rajendra Memorial Research Institute of Medical Sciences (Indian Council of medical Research), Agamkuan, Patna, Bihar, India; 2 Laboratory of Molecular Biochemistry and Cell Biology, Division of Biochemistry, Rajendra Memorial Research Institute of Medical Sciences (Indian Council of medical Research), Agamkuan, Patna, Bihar, India; 3 Division of Molecular Biology, Rajendra Memorial Research Institute of Medical Sciences (Indian Council of medical Research), Agamkuan, Patna, Bihar, India; 4 Division of Immunology, Rajendra Memorial Research Institute of Medical Sciences (Indian Council of medical Research), Agamkuan, Patna, Bihar, India; Institute of Materials Science, GERMANY

## Abstract

Leishmaniasis caused by *Leishmania* parasite is a global threat to public health and one of the most neglected tropical diseases. Therefore, the discovery of novel drug targets and effective drug is a major challenge and an important goal. *Leishmania* is an obligate intracellular parasite that alternates between sand fly and human host. To survive and establish infections, *Leishmania* parasites scavenge and internalize nutrients from the host. Nevertheless, host cells presents mechanism like nutrient restriction to inhibit microbial growth and control infection. Zinc is crucial for cellular growth and disruption in its homeostasis hinders growth and survival in many cells. However, little is known about the role of zinc in *Leishmania* growth and survival. In this study, the effect of zinc on the growth and survival of *L*.*donovani* was analyzed by both Zinc-depletion and Zinc-supplementation using Zinc-specific chelator N, N, N', N'–tetrakis (2-pyridylmethyl) ethylenediamine (TPEN) and Zinc Sulfate (ZnSO_4_). Treatment of parasites with TPEN rather than ZnSO_4_ had significantly affected the growth in a dose- and time-dependent manner. The pre-treatment of promastigotes with TPEN resulted into reduced host-parasite interaction as indicated by decreased association index. Zn depletion resulted into flux in intracellular labile Zn pool and increased in ROS generation correlated with decreased intracellular total thiol and retention of plasma membrane integrity without phosphatidylserine exposure in TPEN treated promastigotes. We also observed that TPEN-induced Zn depletion resulted into collapse of mitochondrial membrane potential which is associated with increase in cytosolic calcium and cytochrome-c. DNA fragmentation analysis showed increased DNA fragments in Zn-depleted cells. In summary, intracellular Zn depletion in the *L*. *donovani* promastigotes led to ROS-mediated caspase-independent mitochondrial dysfunction resulting into apoptosis-like cell death. Therefore, cellular zinc homeostasis in *Leishmania* can be explored for new drug targets and chemotherapeutics to control Leishmanial growth and disease progression.

## Introduction

Leishmaniasis, a neglected tropical disease affecting 350 million people, is prevalent across 98 countries worldwide with higher incidence in tropic and sub-tropical region. Of these, the most severe one, VL has a disease burden of 0.2 to 0.4 million cases with a mortality rate of 20,000 to 40,000 reported per year [1]. *Leishmania donovani* complex is constituted by *L*. *donovani*, *L*. *infantum* and *L*. *archibaldi* in Old world and *L*. *chagasi* in New World [2]. *L*. *donovani*, the causative agent of Indian VL, is an obligate intracellular digenetic protozoan parasite that alternate between an insect and a mammalian host during its life cycle. It harbours inside the sandfly midgut and the macrophages of the mammalian host [3, 4].

In order to survive and establish a successful infection in mammalian host *Leishmania* parasites scavenge and internalize nutrients obtained from the host. Nevertheless, the host presents several mechanisms to control the infection, one of which is nutrient restriction, also known as nutritional immunity [5].

Zinc (Zn), an essential metal, is fundamental for all domains of life. It composes the catalytic and structural centre of large array of proteins that are involved in wide range of physiologic functions including DNA and RNA synthesis, cell growth, protein synthesis, energy metabolism, cellular antioxidant defense, brain development, bone formation, and the immune system function [6,7]. It is present in all six major functional classes of enzymes and catalytically required for the activity of DNA and RNA polymerases [8]. It is also the structural component of many DNA binding transcription regulators where they are required for the proper folding and binding to DNA [9–11]. Cellular zinc content and its distribution is strictly regulated, a prerequisite for its regulatory function. 50% of cellular zinc is localized in the cytosol and cytosolic organelles, 30–40% in the nucleus and remaining is associated with membranes or as free ionic zinc [12]. Intracellular zinc is either tightly bound to proteins, which is known as the non-exchangeable pool of zinc, loosely bound to proteins, or as free Zn^2+^ are collectively known as the labile intracellular pool of zinc (LIPZ) [13]. LIPZ is metabolically important, and the abundance of LIPZ is generally in the femtomolar-picomolar range in many cells [14, 15]. Enzymes or transcription factors can passively acquire Zn from the cytosolic pool. When the zinc quota is reduced, cell growth is stopped and it becomes intoxicated when the zinc burden exceeds an upper threshold level. Interestingly there is a very narrow tolerance range for cytoplasmic Zn and various cells have different machinery to maintain this balance between Zn deficiencies and overload [9, 11]. A lower abundance of LIPZ is associated with impaired DNA synthesis, cell proliferation and in increased apoptosis in many cells [13, 15, 16].

Zinc is also one of the most relevant and an essential nutrient for parasite replication and infectivity for *Leishmania*. Several essential *Leishmania* proteins are known or predicted to bind Zn. The most prominent example is a Zn metalloprotease known as major surface protease (MSP or GP63) which is a virulence factor implicated in several functions along with parasite development [17, 18]. Zinc also plays a structural role in Glyoxalase II an enzyme of glyoxalase pathway that catalyses the formation of the D-lactate from methylglyoxal, a toxic by-product of glycolysis [19, 20]. Like other eukaryotic cells, it is not very surprising that any fluctuation in the availability of Zn (in excess or depleted) in the extra- or intracellular milieu may affect the cell physiology and survival of *Leishmania* parasite.

In case of *Leishmania*, Zn has shown its potential toxic effect against *L*. *major* and *L*. *tropica* [21]. Similarly 1,10-phenanthroline (PHEN), a Zn chelator; has also been shown dose dependent reduction in parasite proliferation in *Trypanosoma cruzi* and *L*. *amazonensis*. PHEN has shown the inhibition of GP63, a Zn-dependent metalloprotease; activity as a mode of killing in *L*. *amazonensis* by their Zn chelating activity as shown by reversal of growth inhibition by addition of Zn [22, 23]. A decrease in association index between PHEN pretreated parasites and mouse peritoneal macrophages have also been reported [23].

However, till date no study has been done on *L*. *donovani* to show the effect of Zn on its growth and survival. Here in this study, we showed that depletion of labile intracellular pool of Zn (LIPZ) as induced by the membrane permeable Zn-specific chelator had more profound effect on the growth inhibition of *L*. *donovani* rather than extracellular Zn depletion or Zn supplementation. Moreover, while investigating for the possible mechanism involved in Zn-depletion induced cell death; we found that Zn depletion induced oxidative stress as characterized by increased reactive oxygen species (ROS). Simultaneously, this increase in ROS caused decrease in total intracellular thiol, depolarization of mitochondrial membrane potential resulting into increase in intracellular calcium and release of cytochrome c into cytosol and DNA degradation leading to apoptosis like cell death. Collectively our study reveals, for the first time, the role of Zn in cell survival of *Leishmania* parasite and that any alteration in intracellular labile Zn level activates stress signals leading to cell death.

## Materials and methods

### Materials

All the reagents and chemicals were purchased from Sigma Aldrich or otherwise mentioned.

### Parasite culture

Standard reference strain AG83 (MHOM/IN/1983/AG83) of *Leishmania donovani* was used in the present study [3]. Parasites were routinely cultured and maintained in M-199 medium containing 100 μg/ml streptomycin, 100 u/mL Penicillin-G and 2 g/L NaHCO_3_ supplemented with 10% fetal bovine serum (Gibco^®^, USA) at 24°C and subcultured after every 72 hr.

### Monocyte culture & differentiation

THP-1 cells, a human leukemic monocytic cell line; were cultured in RPMI 1640 with 10% FBS till 80–90% confluence [3]. Then THP-1 cells were cultured on cover slips in 6-wells plates and treated with 25 nM phorbol 12-myristate 7-acetate (PMA) for 72 hr to adhere and transformed into mature macrophage-like phenotype. After incubation, non-adhered cells were removed by washing with pre-warmed RPMI 1640 without FBS and resuspended in complete RPMI and rested for 24 hr. The THP-1-derived macrophages are now ready for further experiment.

### Cytotoxicity assay

3-(4, 5-dimethylthiazol-2-yl)-2, 5-diphenylterazolium bromide (MTT) assay was preferred to determine the effect of Zn on cell viability of *L*. *donovani* promastigotes as described previously [24]. Log phase *L*. *donovani* promastigotes (1×10^6^ cells/mL) were grown in complete RPMI 1640 media supplemented with 10% FBS in the presence or absence of Zn^2+^ ions by adding different concentrations of intracellular Zn chelator N,N, N', N'- tetrakis (2-pyridylmethyl) ethylenediamine (TPEN) (0–15 μM) extracellular Zn chelator Diethylenetriaminepentacetic acid (DTPA) (0–1.5 mM) and Zinc sulphate (ZnSO_4_) (0–2 mM) for 0–72 hr. At 24 hr interval 10μl of 5 mg/mL MTT solution was added to 100 μl of culture in 96-well microplate and incubated at 24°C for 3 hr. After incubations were over formazan crystals were solubilised by solubilisation solution (acidified isopropanol with triton-X 100) and incubated at 37°C for 30 min. The Optical Density (OD) was recorded on an ELISA reader (iMark Microplate Reader, Bio-Rad, USA) at 570 nm and cell viability was determined. Percent cell viability was determined by dividing OD of treated parasite with untreated parasite multiplied by 100.

### Cell growth

To evaluate the effect of Zn chelation and supplementation on the cell growth, 1× 10^6^ cells per mL were grown in fresh RPMI 1640 medium with and without TPEN and ZnSO_4_. The growth of the parasite was assessed by evaluating the cellular density at 0–120 hr by trypan blue dye exclusion method. Viable cells were quantified by counting the number of non-stained cells using Haemocytometer. Results were expressed as mean±SD from triplicates of three independent experiments.

### Effect of other bivalent ions on the toxicity of TPEN

To evaluate whether the toxicity of TPEN exerted is specifically due to Zn^2+^ ion chelation, promastigotes were treated with different bivalent metal ion salts e.g., ZnSO_4_, CuSO_4_, CaCl_2_, and MgSO_4_ in the presence and absence of 15 μM TPEN for 24 hr. After incubation viability of cells were determined by MTT assay and expressed as percent control viability.

### Analysis of reversibility of the effect of TPEN on parasite proliferation

1×10^6^ *L*. *donovani* promastigotes in the logarithmic phase of growth were treated with 15 μM Zn chelator TPEN and incubated for 24 and 48 hr. After subsequent incubations, parasites were washed twice with PBS, inoculated in the fresh M199 with 10% FBS, and cell density was determined for 0–120 hr at 24 hr interval by counting with a haemocytometer [25]. An additional control of parasites was cultured continuously in the presence of 15 μM TPEN for 72 hr. Three independent assays were carried out in triplicate.

### Morphological analysis of promastigotes

To determine the effects of Zn depletion/supplementation on the morphology of *L*. *donovani* promastigotes, the parasites were harvested by low-speed centrifugation, washed twice with PBS, fixed in 2% formaldehyde, resuspended in PBS and then observed under 40x objective of microscope (Olympus BX41, USA). Images were processed by Image-Pro express software and adobe photoshop element 10.

### Host-parasite interaction study

To determine the effect of Zn chelation on *Leishmania*-macrophage interaction, promastigotes were treated and infected to THP-1 derived macrophages following the method as described by Lima *et al*., 2009 with minor modification [23]. Briefly, stationary phase promastigotes were treated with 7.5 μM TPEN (Zn-depleted) and 100 μM ZnSO_4_ (Zn-supplemented) for 16 hr, washed and resuspended in RPMI 1640 without FBS to maintain the cells in Zn-deficient condition. Viability was checked by mobility and trypan blue staining. THP-1-derived macrophages were then infected for 24 hr with treated and untreated promastigotes at parasite/macrophage multiplicities of 10:1. The unbound parasites were removed by washing with RPMI without FBS. Cover slips were then fixed in absolute methanol, stained with May-Gruenwald giemsa and observed under optical microscope (Olympus, BX41, USA) at 100X with oil immersion. Percent infected macrophage was calculated by counting the number of infected cells as determined by presence of at least single amastigote and parasite load was determined by counting the number of amastigotes per 100 macrophages. Association index was calculated by multiplying the mean number of amastigote per infected macrophage with percent infected macrophages in each group. Each measurement was performed in triplicate and the data was expressed as mean±SD of two independent experiments.

### Measurement of labile intracellular pool of Zn (LIPZ)

A Change in LIPZ in promastigotes was determined following protocol of Haase *et al*, 2008 with minor modifications [26]. Briefly, after individual treatments parasites were harvested and washed thrice with PBS (pH 7.4). Cells were then incubated with 1 μM FluoZin-3AM (Invitrogen, USA) in incubation buffer (25 mM HEPES (pH 7.35), 120 mM NaCl, 5.4 mM KCl, 1.3 mM CaCl_2_, 1 mM MgCl_2_, 1 mM NaH_2_PO_4_, 5 mM Glucose, 0.3% BSA) for 30 min at room temperature. Subsequently, cells were washed twice with measurement buffer (incubation buffer without BSA) and transferred into a 96-well plate at a density of 2×10^6^ cells/ml. The resulting fluorescence was recorded on a fluorescence well plate reader (Agilent technologies, USA) at excitation wavelength of 485 nm and emission at 535 nm. Promastigotes treated with pyrithione/ZnSO_4_ (50/100 μM) and TPEN 50 μM was used to serve as positive and negative control to measure maximum & minimum LIPZ. Changes in the amount of fluorescence detected were considered as change in the LIPZ.

### Measurement of reactive oxygen species

To measure reactive oxygen species (ROS) treated and untreated parasites were harvested at each time points, washed twice with PBS and resuspended in RPMI (without FBS) with 50 μM cell permeable oxidative fluorescent dye 2', 7' dichlorodihydrofluorescein diacetate (H_2_DCFDA) (Sigma Aldrich, USA) and incubated for 30 min at 37°C. ROS was estimated by measuring fluorescence intensity at excitation and emission wavelength of 504 and 529 nm respectively using spectrofluorimeter (Agilent Technologies, USA) as described previously [3]. The fluorescence intensity is directly proportional to ROS generated. N-acetyl cysteine (NAC), a ROS scavenger, was used as control.

### Determination of reduced thiol content

The total intracellular reduced thiol level was measured in deproteinized cell extracts from treated and untreated *Leishmania* parasites by the method as described elsewhere with slight modifications [27]. Briefly, the cells were harvested and washed twice with PBS buffer (pH-7.4). Cell pellet was lysed and deproteinized by adding 25% trichloroacetic acid. Cell lysate was then centrifuged to remove denatured protein and cell debris and supernatant was collected. Thiol content in the supernatant was determined spectrophotometrically at 412 nm as 5, 5’-dithio-bis (2-nitrobenzoic acid) (DTNB) derivatives of thiols with 0.6 mM Ellman’s reagent (DTNB) in 0.2 M Sodium phosphate buffer, pH 8.0. Data were expressed as mean±SD of three independent experiments in triplicates.

### Semi-quantitative RT-PCR

Zn-depleted and Zn-supplemented parasites were harvested after 16 hr of incubation. RNA was extracted by using RNeasy^®^ Plus Mini Kit (QIAGEN) and c-DNA was prepared from 2 μg of total RNA using QuantiTect^®^ Reverse transcription kit (QIAGEN) as per manufacturer’s protocol. The c-DNAs were then amplified by PCR for thiol metabolic pathway genes using gene specific primers as follows: for gamma-glutamyl cysteine synthetase (γ-GCS), 5’- AGCGATAAACCGCTCGTACTGTGA-3’ (F), 5’- ATGTTGTCAAAGTGCTCCGTGTGC-3’ (R); for trypanothione synthetase (TryS), 5’- TGTCATGAGCGAATGACCAACCGAT-3’ (F), 5’- GCTTGCCATTCAACAAACGTCAGGT-3’ (R); for trypanothione reductase (TR), 5’- AATGAGGACGGCTCGAATCACGTT-3’ (F), 5’- ATGGCGTAGATGTTGTCCACCGAT-3’ (R) using initial denaturation at 94°C for 5 min and 25 amplification cycles (94°C for 30 s, 55°C or 58°C for 30 s, and 72°C for 1 min), followed by a final extension at 72°C for 5 min [28]. The products were run on 1.5% agarose gel, stained with ethidium bromide prior to analysis, and visualized and quantified using the Bio-Rad gel documentation system and associated Quantity One software. Alpha-tubulin (α-tubulin) was used as house-keeping gene. Each measurement was performed in triplicates and data was expressed as mean±SD of three independent experiments.

### Annexin-V binding assay

Annexin V-FITC staining was performed by Annexin-V-FITC kit (Cayman Chemical, USA) as per manufacturer’s instructions. Briefly, after individual incubations, treated and untreated *Leishmania* parasites were harvested, washed twice in PBS (pH 7.2), resuspended in Annexin-V binding buffer followed by addition of Annexin V-FITC and Propidium Iodide (PI). The cells were then incubated in the dark at 25°C for 15 min, washed once with binding buffer and acquired on BD FACS Calibur flow cytometer followed by analysis with Cell Quest software. Parasites showing Annexin V^+^ PI^-^ was considered as early apoptotic, Annexin V^+^ PI^+^ late apototic, Annexin V^-^ PI^+^ necrotic and Annexin V^-^ PI^-^ as healthy cells.

### Measurement of mitochondrial membrane potential (Δψm)

Changes in mitochondrial membrane potential (Δψm) in *Leishmania* promastigotes was estimated using Tetramethyl rhodamine ethyl ester perchlorate (TMRE) (Sigma Aldrich, USA) dye as described previously by Mesquita *et al*, 2013 with minor modifications [25]. Briefly, after 24 hr of incubation, treated and untreated *Leishmania* parasites were harvested, washed with PBS and incubated with 50 nM TMRE dye suspended in RPMI 1640 without FBS for 30 min at RT. After incubation cells were harvested and resuspended in PBS with 0.2% bovine serum albumin and analyzed by BD FACSCalibur flow cytometer at FL-2 channel. A total of 10,000 events were acquired in the region established for parasites. Index of variation (IV) was calculated by equation (MT—MC/MC), where MT and MC are the mean fluorescence intensity of treated and untreated control parasites respectively to quantify alterations in TMRE fluorescence. Depolarization of the mitochondrial membrane was defined by negative IV and hyperpolarization by positive IV values. 10 mM carbonyl cyanide 4-(trifluoromethoxy) phenylhydrazone (FCCP), was used as a positive control which dissipates the Δψm.

### Measurement of intracellular calcium level

Intracellular Calcium concentration was measured by using the fluorescent probe Fura-2-AM (Sigma Aldrich, USA) as described previously by Sardar *et al*, 2013 [29]. Ethylene glycol-bis(2-aminoethylether)-N,N,N’,N’-tetraacetic acid (EGTA), a calcium chelator was used as control. Each measurement was performed in triplicate and the data were expressed as mean±SD for three independent experiments.

### Immunoblot analysis

*Leishmania* parasites were harvested after 16 hr of incubation and washed once with PBS. Cytosolic and mitochondrial fractions were isolated by the method as described earlier by Gannavaram *et al*., 2008 with minor modifications [30]. Briefly, *Leishmania* promastigote cells were washed thrice in MES buffer (20 mM MOPS, pH 7.0, 250 mM Sucrose, 3 mM EDTA) and resuspended in 200 μl MES buffer containing 0.25 mg ml^-1^ digitonin along with 1X protease inhibitor cocktail. The suspension was vortexed vigorously, incubated at room temperature for two minutes and then centrifuged at 10,000 g for 5 min at 4°C to separate mitochondrial fraction (in pellet) from the cytosol (supernatant). The mitochondrial-enriched pellet fraction was then washed with MES buffer and resuspended in 100 μl MES buffer containing 0.25 mg ml^-1^ digitonin, 1% Triton X-100 along with 1X protease inhibitor cocktail incubated for 5 min and then centrifuged at 10,000 g at 4°C for 5 min, clear supernatant was separated as mitochondrial protein fraction. For confirmation of the semi-quantitative RT-PCR data, 30 μg cytosolic fractions collected from Zn-depleted and Zn-supplemented parasites were separated on 12% SDS-polyacrylamide gel, immunoblotted and probed with rabbit polyclonal anti-TryS (1:3000) and anti-TR (1:2000) antibodies [31]. For cytochrome-c detection, the cytosolic fractions of all the treated and untreated control parasites were analysed by immunoblotting using rabbit polyclonal anti-cytochrome c antibody (Santa cruz Biotechnology, USA) (1:1000). Alkaline phosphatase-conjugated secondary antibody (1:10000) was used in all cases and β-actin (β-act) was used as an endogenous control. The protein band was visualized by NBT-BCIP staining and expressed as band intensity.

### Caspase activity assay

To determine the caspase activity of *Leishmania* parasites, a fluorogenic caspase assay was performed using the Caspase DEVD-RC110 substrate (Cayman Chemical, USA) as per manufacturer’s instructions. The fluorometric measurement was recorded with a spectrofluorimeter (Agilent Technology, USA) with excitation at 485 nm and emission at 535 nm.

### DNA fragmentation assay

DNA fragmentation was assessed by using Cell Death Detection ELISA plus kit (Roche Applied Science, Germany) as per manufacturer’s instruction. Briefly, after individual incubations both treated and untreated parasites were harvested and lysed in lysis buffer. The cell lysates were added streptavidin-coated wells and incubated with biotin-conjugated mouse monoclonal anti-histone antibody and peroxidase-conjugated mouse monoclonal anti-DNA antibody. ABTS [2,2’-azino-bis(3-ethylbenzthiazoline-6-sulphonic acid)] was used as a colour developing reagent to estimate mono- and oligonucleosomes in the cytosolic fractions generated due to DNA fragmentation. Absorbance was measured at 405 nm with a reference wavelength of 490 nm in a Thermo Multiskan EX plate reader. Results were expressed as relative percentages.

### Statistical analysis

All results were shown as mean±standard deviation (SD) from three independent experiments done in triplicates. Statistical analysis was performed using GraphPad Prism Program (Version 5.0, GraphPad Software, USA). In vitro antileishmanicidal activity, expressed as IC_50_, was determined by non-linear regression analysis. Statistically significant differences among different groups were determined by Student’s t test or Kruskal-Wallis Test and Dunn’s multiple comparison tests. P–value ≤ 0.05 were considered significant.

## Results

### Zn is crucial for growth and survival of *Leishmania donovani* promastigotes

Zinc plays an important role in several biochemical processes, structural activities and metabolic pathways including gene expression regulation and cell proliferation. Zinc may serve as a regulator of cell fate and its extra- and intracellular level determine if the cell will die or proliferate [32]

**Fig 1:**
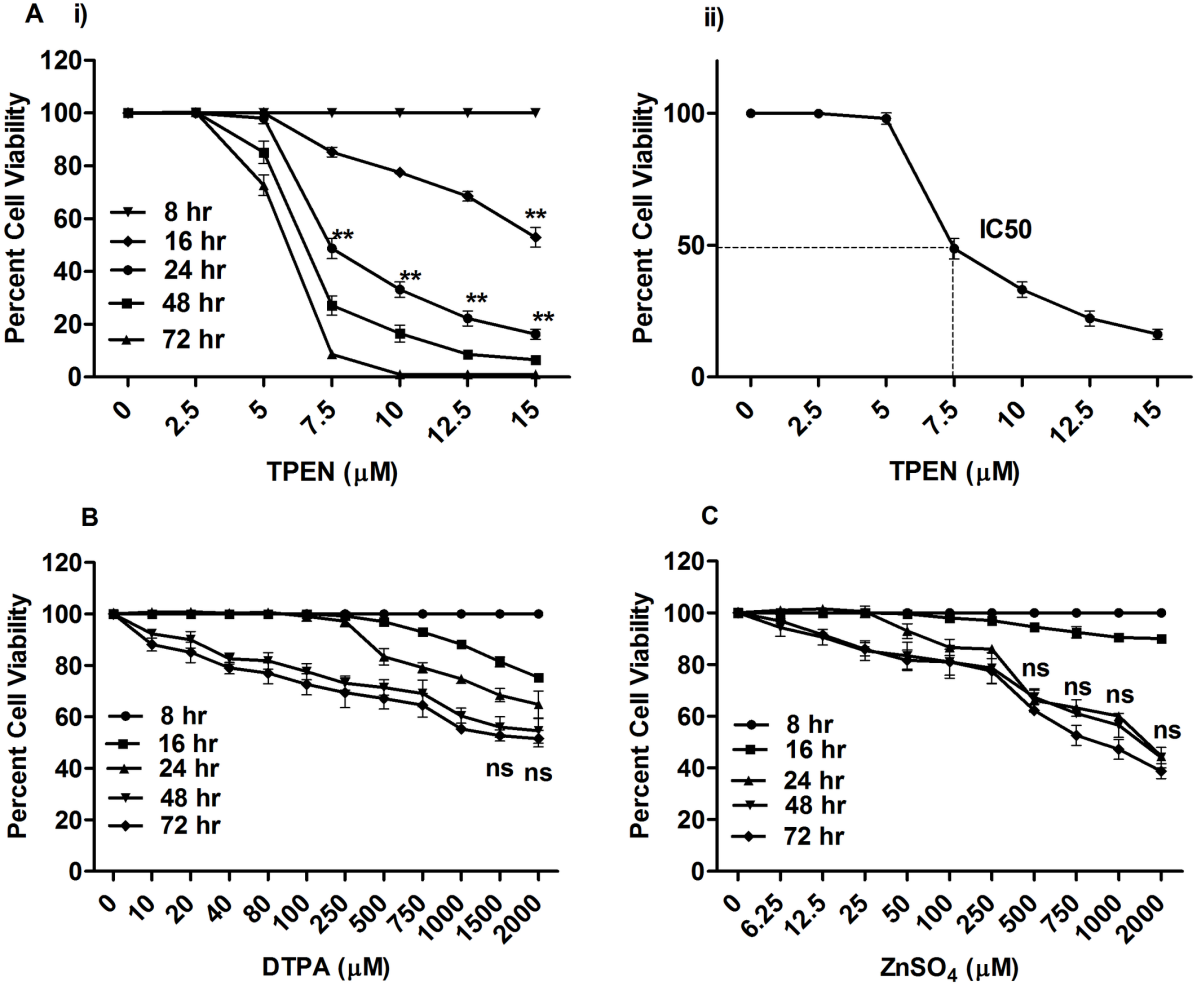
Effects of TPEN, ZnSO_4_ and DTPA on the viability of *L*. *donovani* promastigotes. Parasites were grown in the presence and absence of TPEN (A), DTPA (B) and ZnSO_4_ (C) in RPMI 1640 medium supplemented with 10% fetal bovine serum and percent cell viability was determined at 8, 16, 24, 48, 72 hr by MTT assay. The data represent mean±S.D. from three independent experiments in triplicates. IC_50_ value in TPEN treated parasites were determined by non-linear regression analysis (A ii). * P< 0.05, **P< 0.001, ns Not significant; as compared between different time points in treated promastigotes by Student’s t test.

To determine the effect of Zn in *L*. *donovani* promastigotes, Zn level was altered by either depleting it by adding cell permeable zinc chelator TPEN or cell impermeable chelator DTPA or supplementing the culture medium with zinc in the form of ZnSO_4_. Promastigotes were incubated at different concentrations for 0–72 hr; cell viability was assessed by MTT method and calculated as percent of untreated control. Both Zn depletion and Zn supplementation resulted into dose and time dependent decrease in cell viability (Fig 1A–1C). However, significant cell death was observed when intracellular Zn was depleted by using TPEN (P<0.001). The IC_50_ for TPEN was found to be 7.5 μM at 24 hr (Fig 1A(ii)). In comparison to intracellular Zn depletion, the extracellular Zn chelation by DTPA showed lesser effect on cell viability. Reduced growth of *Leishmania* parasite by Zn supplementation was also observed at higher dose as 1 mM was calculated as the IC_50_ dose. The lower physiological doses of ZnSO_4_ enhanced the cell survival of parasites. From these observations it is evident that alteration in intracellular Zn level caused more pronounced decrease in *Leishmania* viability than extracellular Zn-depletion or supplementation. To elucidate the mechanism of effect of Zn in *Leishmania*, three doses of TPEN viz. 5, 7.5 & 15 μM was selected as treatment doses and 16 & 24 hr as time point for further experiments.

### Zn chelation arrests cell growth and Zn supplementation restores the growth in Zn depleted parasites

**Fig 2:**
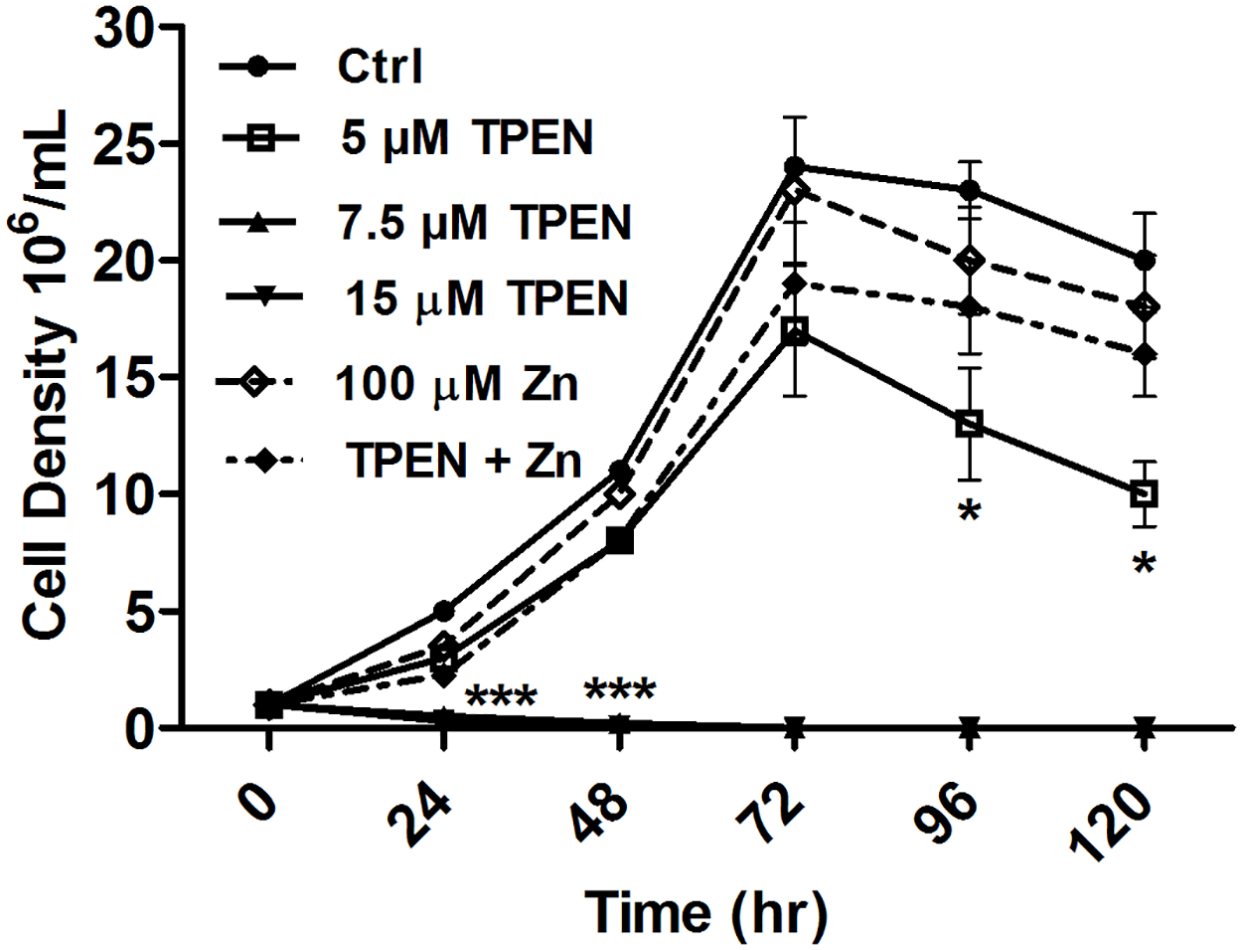
Zinc depletion reduced the cell growth in *L*. *donovani* promastigotes. Parasites were cultivated in M199 medium supplemented with 10% fetal bovine serum in the presence or absence of TPEN or ZnSO_4_ for 0–120 hr. Cell proliferation was checked by cell counting daily at 24 hr interval. Bars represent mean±S.D. from three independent experiments. * P< 0.05, **P< 0.001, ***P<0.0001, ns Not significant; as compared between different time points in treated promastigotes by Student’s t test.

To determine the effect of TPEN & ZnSO_4_ on the cell proliferation and cell growth, parasites were treated with different concentrations of TPEN and ZnSO_4_ and cell density was measured at 24 hr interval by counting in haemocytometer chamber. In presence of 5 μM TPEN, promastigotes proliferation was found to be comparatively lower at all time points, whereas in the presence of Zn^2+^ ions alone or in combination with Zn chelator cell proliferation was similar to the untreated control (Fig 2). However, promastigotes growth was drastically reduced at 24 hr at 7.5 μM and 15 μM of TPEN concentration (p<0.0001).

**Fig 3:**
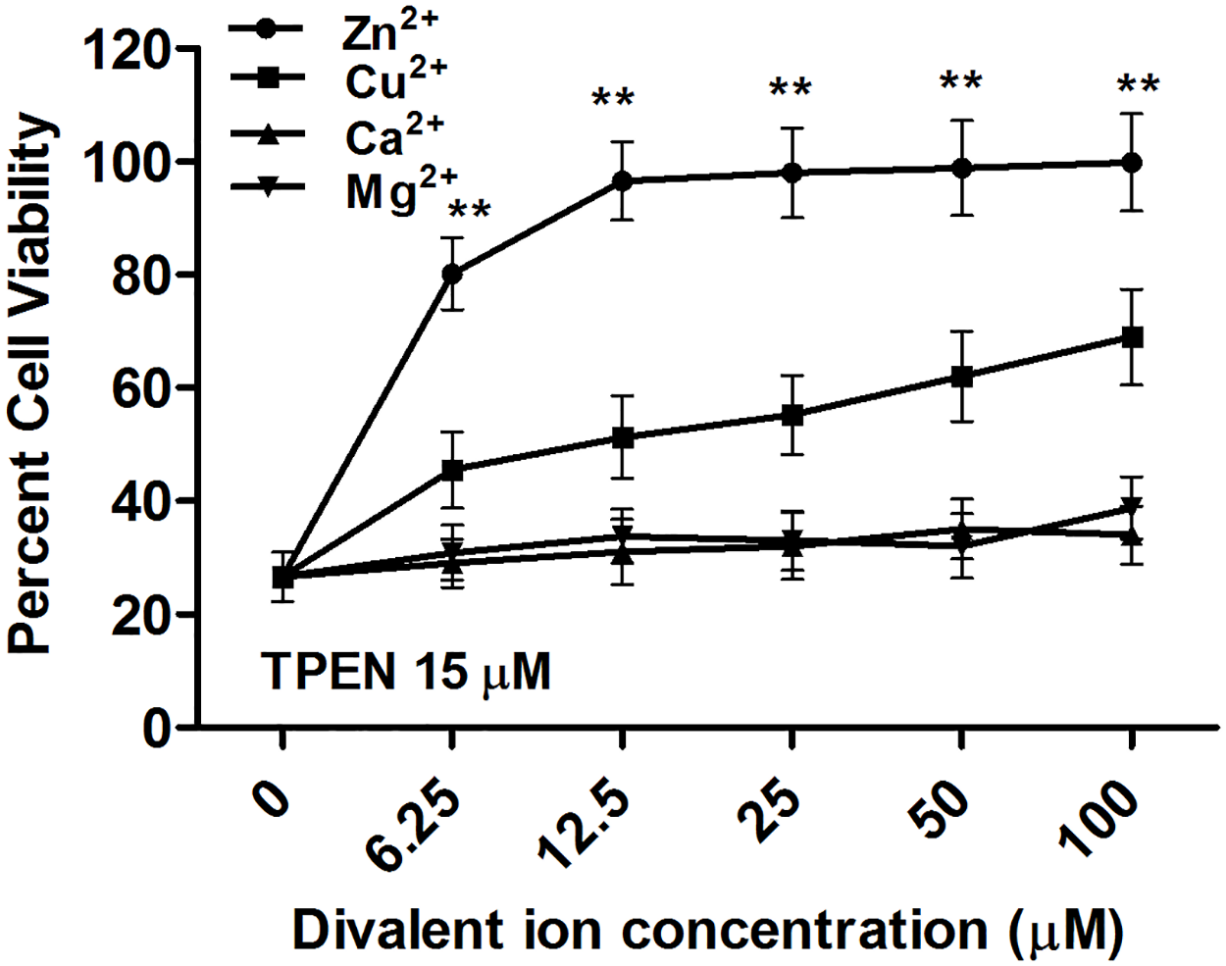
Zinc, not other divalent metal ions; inhibited cytotoxicity of TPEN. *L*. *donovani* promastigotes were treated with 15 μM TPEN in the presence or absence of indicated divalent metal cations for 24 hr and cell viability was measured by MTT assay. Results are expressed as cell viability compared to untreated control and represent mean ± S.D. of three independent experiments in triplicates. * P< 0.05, **P< 0.001, ns Not significant; as compared in divalent metal ions treated and untreated promastigotes by Student’s t test.

To evaluate whether the cell death induced by TPEN is specifically due to Zn chelation not due to other divalent metal ions or non-specific toxicity by TPEN, we treated parasites with 15 μM TPEN in the presence or absence of different concentrations of divalent metal ions viz. Zn^2+^, Cu^2+^, Ca^2+^, Mg^2+^ for 24 hr and cell viability was assessed by MTT assay. A significant increase in cell viability (p< 0.001) was observed when the cells were co-incubated with Zn^2+^. Interestingly Cu^2+^ partially restored parasite growth whereas Ca^2+^ & Mg^2+^ failed to restore the growth in the presence of TPEN (Fig 3). Thus, the results showed that TPEN triggered cell death in *L*. *donovani* were mainly due to the disturbances in intracellular Zn bioavailability.

### Parasite proliferation recuperated after treatment with TPEN

**Fig 4:**
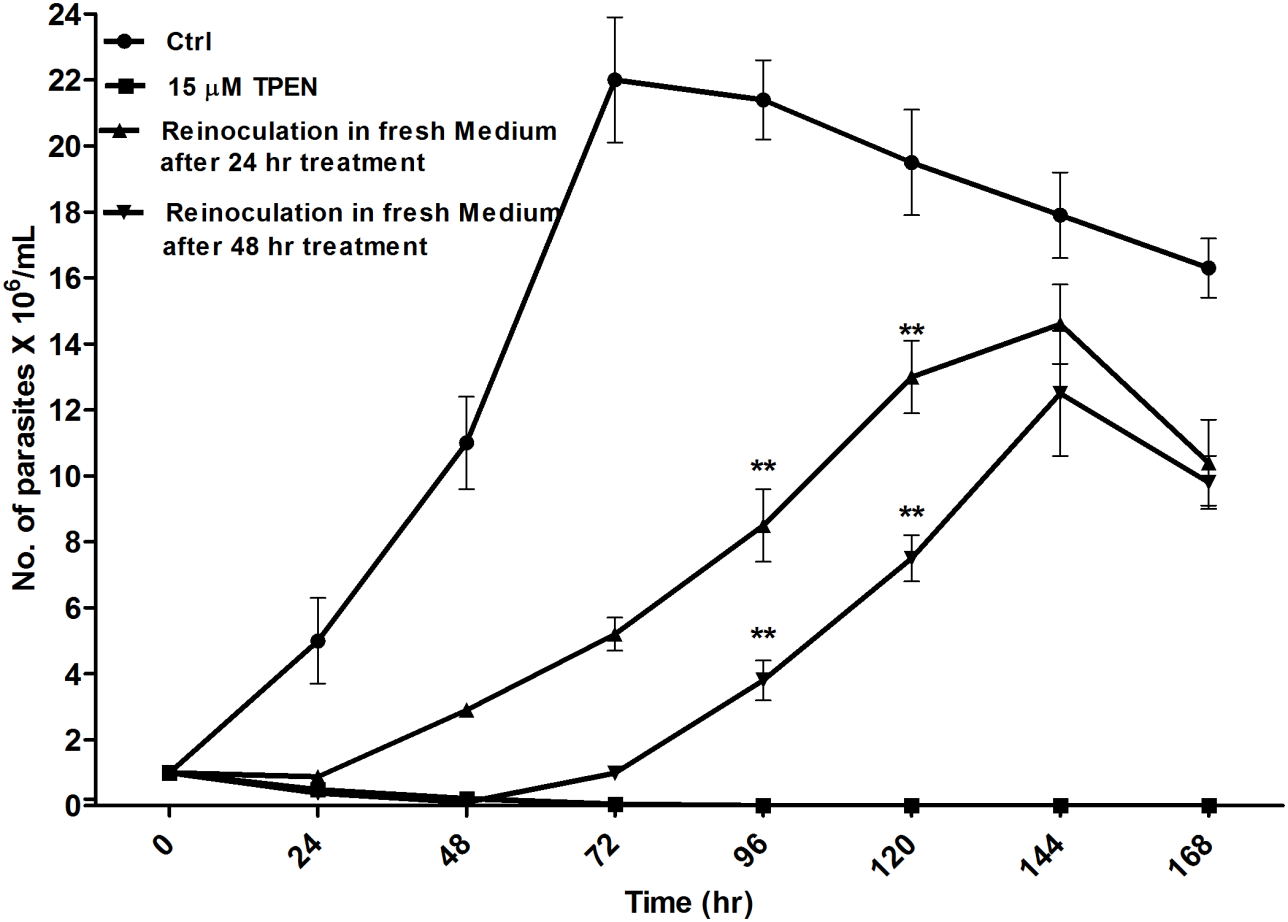
Reversibility in proliferation of *L*. *donovani* after TPEN treatment. Parasites were treated with 15 μM TPEN for 24 and 48 hr and then re-inoculated into fresh medium. Cell density was determined at 24 hr interval by counting with haemocytometer. Cell proliferation in untreated and treated parasites was also monitored. Bar represents mean and standard error of three independent experiments. * P< 0.05, **P< 0.001, ns Not significant; as compared between different time points in treated promastigotes by Student’s t test.

To determine whether the proliferation capacity of *L*. *donovani* could be recovered after intracellular Zn deprivation, parasites treated with 15 μM TPEN for 24 hr & 48 hr were re-inoculated in fresh M199 medium with 10% FBS and cell density was monitored daily for 120 hr. Promastigotes treated with TPEN for 24 and 48 hr recovered their proliferation ability after being placed in fresh medium (Fig 4). However TPEN treated parasites showed slightly retarded proliferation during the early stage of growth probably reflecting their re-adaptation period, *L*. *donovani* regain their proliferation ability in fresh medium and more time was taken by treated parasites to reach their stationary phase of growth than untreated control parasites.

### Zinc depletion alters morphology in *L*. *donovani* promastigotes and also affects their interaction with host macrophages

**Fig 5:**
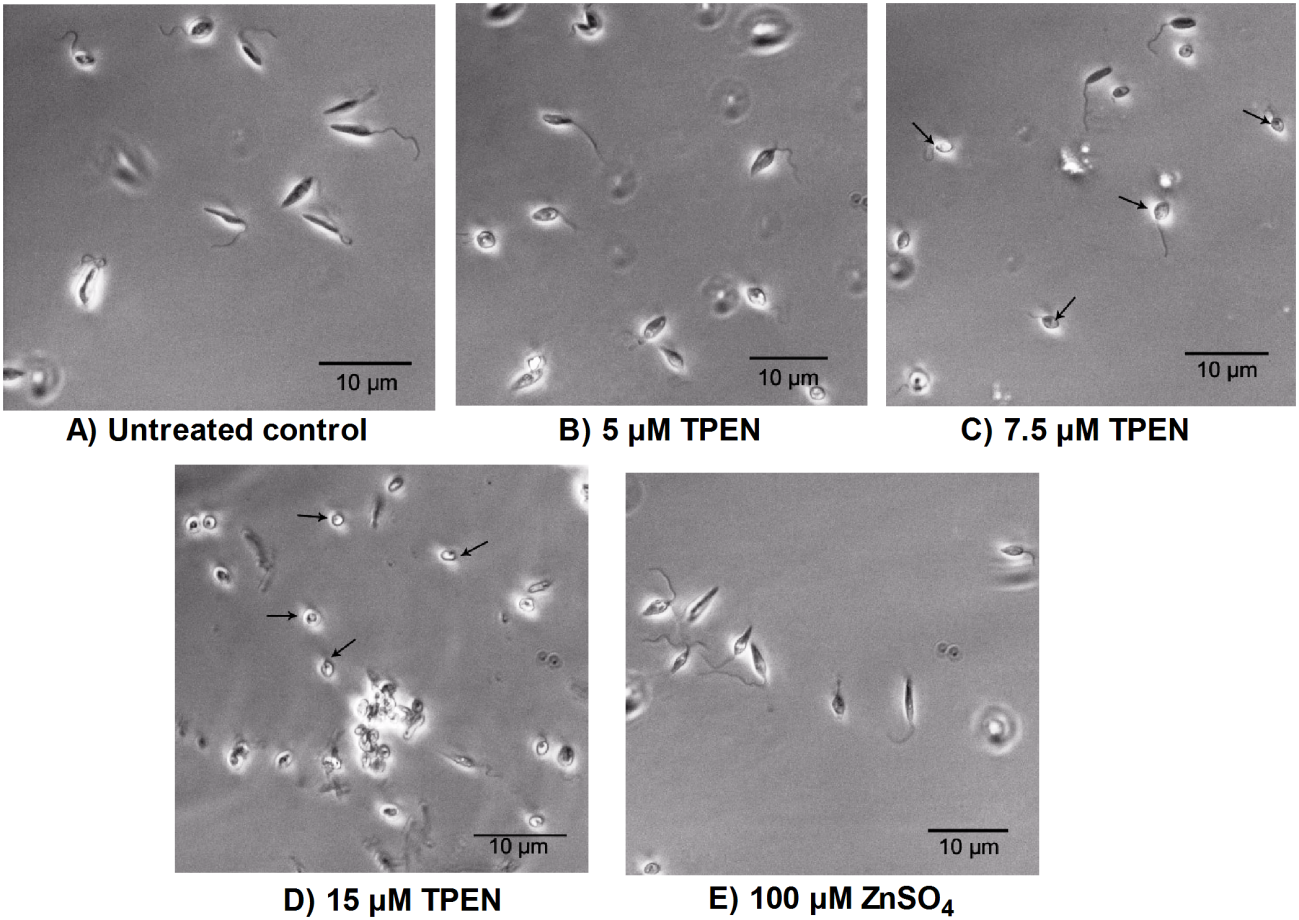
Morphological analysis of treated and untreated parasites. Parasites were cultivated in M199 medium supplemented with 10% fetal bovine serum in the presence or absence of TPEN or ZnSO_4_ for 24 hr. Cell morphology was analysed at 400X by optical microscopy as described in material and method section. Arrow indicates altered morphological forms in *L*. *donovani* promastigotes.

Distinct morphological changes were observed on treatment with higher doses of TPEN. Parasites treated with 7.5 μM and 15 μM of TPEN were substantially reduced in size, became ovoid shaped with cell shrinkage and shortened flagella as compared to untreated control (Fig 5A, 5C and 5D). However, no change in morphology was observed after incubating promastigotes in 5 μM TPEN and 100 μM ZnSO_4_ supplemented medium (Fig 5B and 5E).

**Fig 6:**
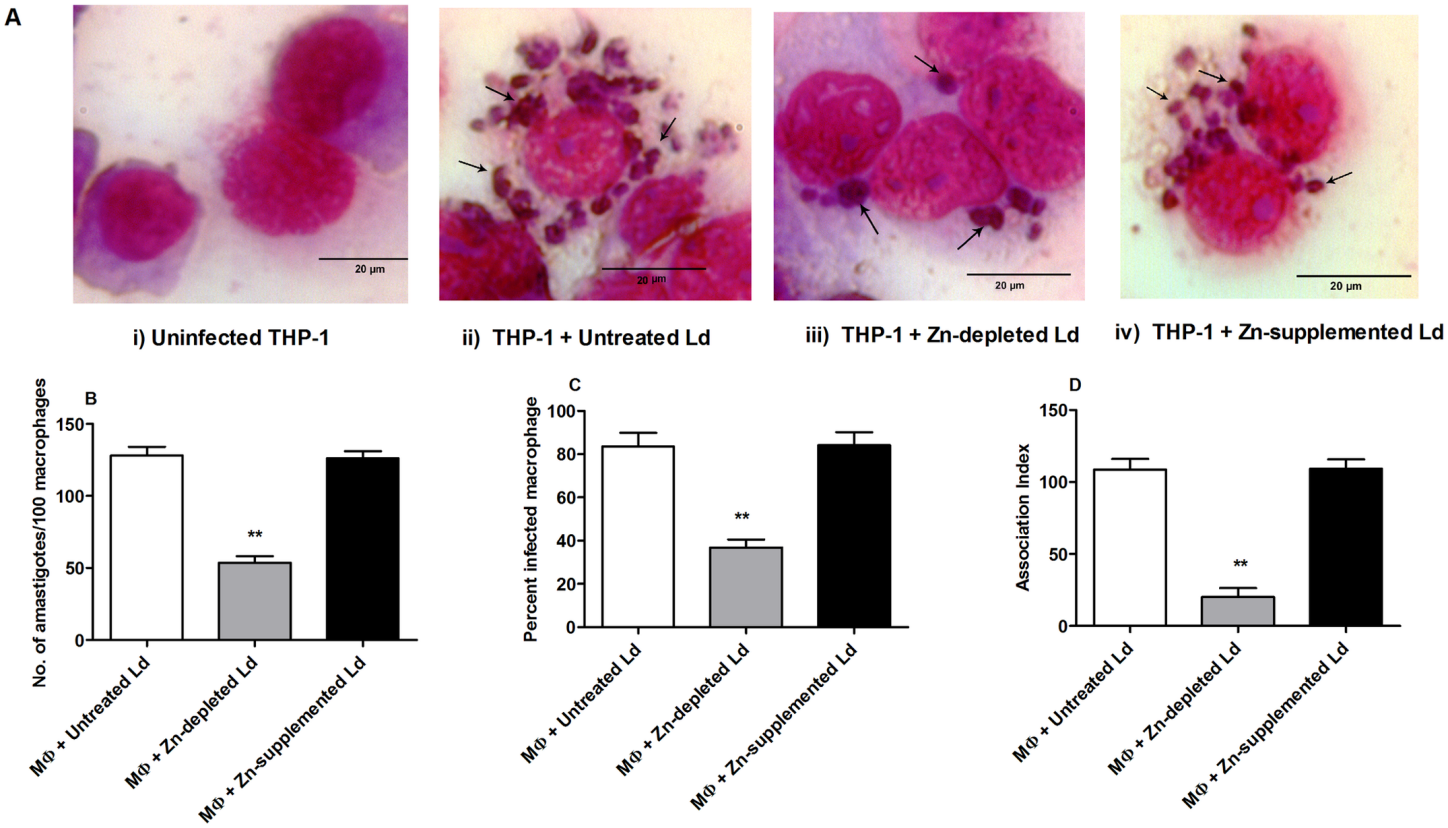
Intracellular zinc depletion decreased interaction between *L*. *donovani* and host macrophages. THP-1 derived macrophages were infected with treated or untreated control parasites for indicated time period and intracellular amastigotes were visualized by giemsa staining followed by optical microscopy at 100x (A). The parasite load in infected macrophages was measured by counting the numbers of intracellular amastigotes per 100 macrophages (B). Rate of infection was determined by counting the number of infected macrophages per 100 macrophages (C). Association index was determined by multiplying mean number of amastigotes with percent infected macrophages (D). The data represents Mean±S.D of three independent experiments in duplicates. * P< 0.05, **P< 0.001, ns Not significant; as compared in zinc depleted and zinc supplemented parasites with untreated control parasites by Kruskal-Wallis test with Dunn’s multiple comparison test. Arrow indicates intracellular amastigotes in macrophage.

Gp63 is a Zinc-dependent metalloprotease, abundantly found on the surface of *Leishmania* parasite. It aids the parasite to interact and adhere with host macrophages and thus helps in successful invasion of host cell [17]. To evaluate the effect of Zn^2+^ depletion and supplementation on the interaction between *Leishmania* parasite and host macrophages, we treated parasites with TPEN (7.5μM; Zn-depleted) and ZnSO_4_ (100 μM; Zn-supplemented) for 16 hr and infected THP-1 derived macrophages for 24 hr (Fig 6A). The sole objective of treating parasites before infecting macrophages was to check whether the Zn^2+^ has any effect on the virulence of the parasites. TPEN, an intracellular Zn^2+^-chelator promoted a decrease in interaction between *L*. *donovani* promastigotes and THP-1 derived macrophages. It was found that the parasite load was decreased by ~ 2.1-fold in macrophages infected with Zn-depleted parasites compared to control (P<0.001) (Fig 6B). However, no significant change was seen in parasite load in macrophages infected with Zn-supplemented parasites. Simultaneously, percent infectivity was also decreased in case of macrophages infected with TPEN treated parasites (P<0.001) (Fig 6C). Consequently, Zn-depletion resulted into decrease in the association index between TPEN treated parasites and host macrophages. Conversely, treatment of parasites with ZnSO_4_ had no significant effect on the interaction of parasites with macrophages. Furthermore, the Zn^2+^-treated parasites showed an association index similar to the control parasites and approximately ~ 4.5-fold higher than TPEN treated parasites (P<0.001) (Fig 6D). Therefore, our results showed that alteration in Zn level regulate the virulence of *Leishmania* parasite as indicated by decreased association index in Zn-depleted parasites compared to Zn-supplemented and control.

### Treatment of parasites with Zn chelator depleted labile intracellular pool of zinc (LIPZ)

The importance of Zn ions in cell proliferation, differentiation and survival is well documented [32]. Considering the inhibition of cell growth by zinc depletion and cell survival & proliferation by zinc supplementation in *Leishmania* parasite, the mechanism of intracellular Zn depletion-induced cell death was investigated.

**Fig 7:**
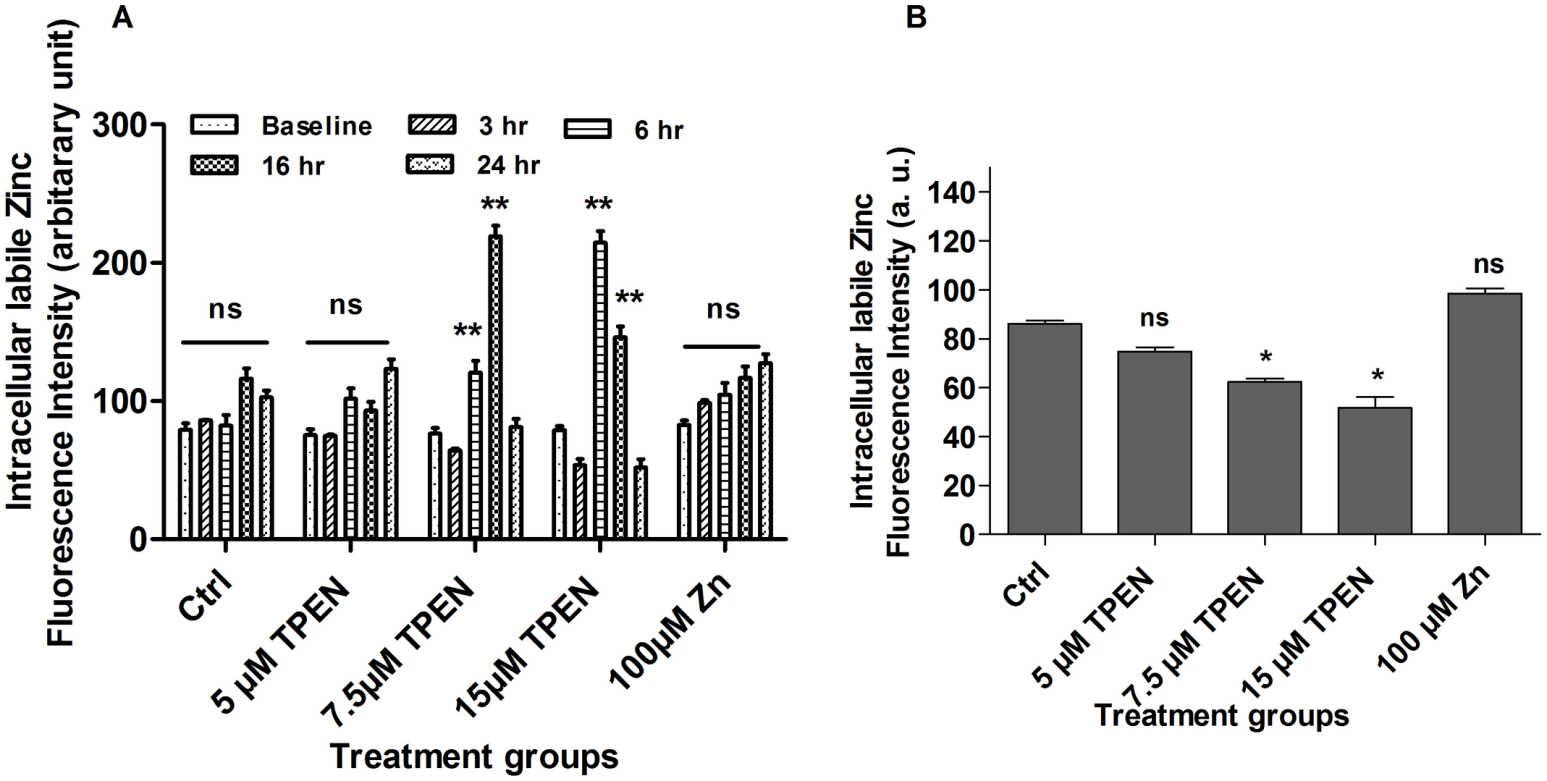
TPEN treatment resulted into flux of LIPZ in dose- and time- dependent manner. Zinc depleted and zinc supplemented *L*. *donovani* was cultivated in RPMI medium for indicated time periods and intracellular labile zinc pool was measured by spectrofluorometry using Fluozin-3AM dye as described in “Materials & methods” section. The data represents Mean±S.D of three independent experiments in triplicates. * P< 0.05, **P< 0.001, ns Not significant; as compared in zinc depleted and zinc supplemented parasites with untreated control parasites by Kruskal-Wallis test with Dunn’s multiple comparison test.

The labile intracellular pool of Zn^2+^ was assayed using Zn-specific lipid soluble, cell permeable fluorescent probe Fluozin-3AM in the form of an acetoxymethyl (AM) ester derivative. This probe forms a 1:1 complex by high affinity binding with Zn^2+^ yielding a good correlation between the labile Zn^2+^ concentration and fluorescence [33]. LIPZ was measured by treating parasites at different doses from 0–24 hr. The results showed dose and time dependent flux in intracellular Zn level in TPEN treated parasites (Fig 7A). LIPZ was reduced in first 0–3 hr as shown by decreased fluorescence intensity of Fluozin-3AM (Fig 7B) after that increase in LIPZ was observed till 16hr and then Zn level was reduced drastically in 24 hr. This flux in LIPZ was also dose dependent as evidenced by increase and decrease in fluorescence intensity. At IC_50_ (7.5 μM) and 2 × IC_50_ (15 μM) doses fluorescence intensity of Fluozin-3AM was both increased and decreased at different time period (P<0.001). At TPEN concentrations of 5.0 μM, 7.5 μM and 15 μM there was ~ 1.4, ~ 2.6 and ~ 2.2 folds increase in fluorescence intensity at 24, 16 and 6 hr respectively. However no such fluctuation was observed in Zn supplemented parasites however an increase in LIPZ was observed after 16 hr incubation. Thus the above observations showed that TPEN treatment resulted into depletion of intracellular zinc in *L*. *donovani* promastigotes.

### Intracellular Zn depletion increases ROS and downregulates expression of trypanothione synthetic enzymes in *Leishmania* parasite

**Fig 8:**
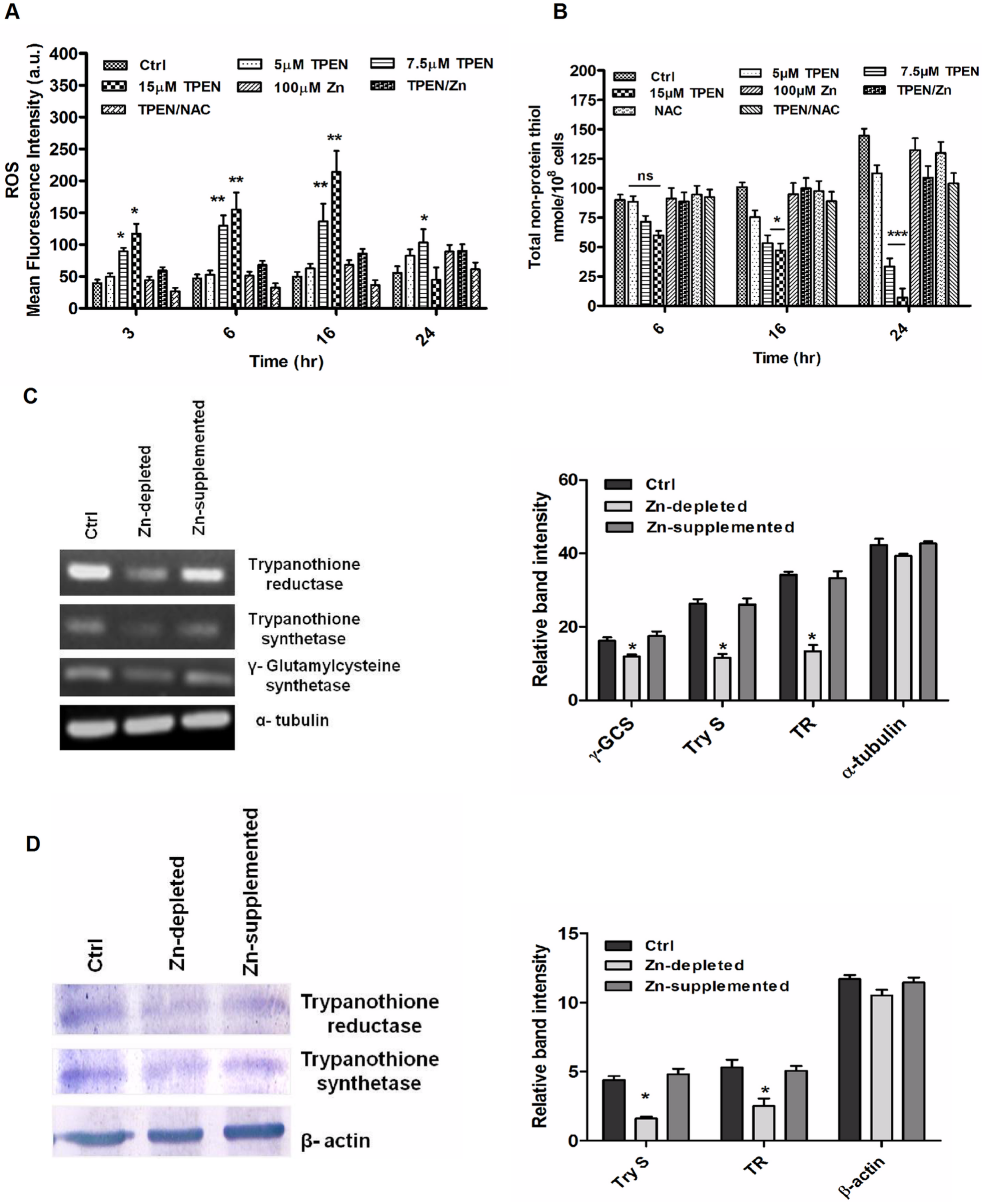
Zinc depletion increases ROS level and downregulates the expression of thiol synthetic pathway enzymes in *L*. *donovani* promastigote. Treated and untreated control *Leishmania* parasites were grown in RPMI media for 0–24 hr. Intracellular ROS was estimated using 2,7-dichlorodihydrofluorescein diacetate (H_2_DCFDA) by spectrofluorometry as described in “Materials & methods” (A). The total intracellular level of reduced thiol was estimated spectrophotometrically using DTNB (B). Expression of key enzymes involved in trypanothione synthesis was analysed by semi-quantitative PCR. Cells were harvested, total RNA was extracted, cDNA was prepared and expression of γ-GCS, Try S, TR and α-tubulin were analysed by semi-QRT PCR using specific primers. The amplicons were visualized on ethidium bromide-stained 1.5% agarose gel and photographed in Gel documentation system (C). Cells were harvested; cytosolic fractions were separated by method as described in “materials and methods”. The cytosolic fractions were separated on SDS-polyacrylamide gel, immunoblotted and probed with rabbit polyclonal anti-leishmania Try S, TR and β-actin antibodies (D). Relative expressions of enzymes at transcriptional and translational level were analyzed by densitometry (C-D). The data represents Mean±S.D of three independent experiments in triplicates. * P< 0.05, **P< 0.001, ns Not significant; as compared in zinc depleted and zinc supplemented parasites with untreated control parasites by Kruskal-Wallis test with Dunn’s multiple comparison test.

It is reported that zinc deficiency has been associated with oxidative stress in different cells [34]. To evaluate the effect of intracellular zinc depletion on generation of oxidative stress in parasites, a cell permeable fluorescent dye H_2_DCFDA was used. This dye upon cleavage by non-specific esterases forms a cell impermeable non-fluorescent dihydrodichlorofluorescein (H_2_DCF) which after being oxidized by intracellular ROS forms a fluorescent compound dichlorofluorescein (DCF). The fluorescence thus generated is directly proportional to ROS level inside the cells. In our study parasites were treated with different doses of TPEN & Zn for 0–24 hr and ROS level was measured. Similar to LIPZ, a dose- and time-dependent flux in intracellular ROS level was observed in zinc depleted parasites when compared to zinc supplemented parasites and untreated control cells. An elevation of ~ 2.7-fold and ~ 4.3-fold ROS was observed in 7.5 μM and 15 μM TPEN treated parasites up to 16 hr (P<0.001) thereafter ROS level was reduced (Fig 8A). However when zinc depleted parasites were treated with a ROS scavenger, NAC, the ROS level was reduced to that of control level. Similarly, when zinc chelator treated parasites were co-incubated in the presence of Zn^2+^, no significant increase in ROS level was observed. This result indicates that increased ROS generation due to intracellular zinc depletion might be a reason for reduced viability for Zn-depleted parasites.

Further, non-protein thiols are important antioxidants which help in maintaining the cellular redox balance [35]. A gradual decrease in thiol levels was observed in Zn-depleted parasites (~ 1.8 & ~ 4.3 fold for 7.5 μM and ~ 2.1 & ~ 7.8 fold for 15 μM TPEN after 16 (P<0.05) & 24 hr (P<0.0001) respectively) with increasing time interval, whereas slight increase in thiol level was observed in Zn-supplemented and untreated control parasites (Fig 8B). This decrease in intracellular thiol in Zn-depleted parasites correlates with increased ROS production leading to decreased parasite survival.

Trypanothione [T(SH)_2_], a dithiol, is the major reduced thiol in *Leishmania* parasites essential in defense mechanism against oxidative stress and thus helps in survival of the parasite [35]. To validate the above findings we further investigated the expression of enzymes γ-GCS, Try S and TR involved in trypanothione biosynthesis. Transcript abundance of γ-GCS (~ 2.0 fold), Try S (~ 2.2 fold) and TR (~ 2.5 fold) were significantly decreased in Zn-depleted parasites as compared to Zn-supplemented and untreated control parasites (P<0.05) as assessed by semi-quantitative RT PCR (Fig 8C).

The expression of Try S and TR was also evaluated at the translational level by immunoblot analysis. A significant decrease in protein level of Try S (~ 2.7 fold) and TR (~ 2.3 fold) was observed in Zn-depleted parasites compared to Zn-supplemented parasites (P<0.05) (Fig 8D).

### Zinc depletion failed to induce phosphatidylserine exposure

**Fig 9:**
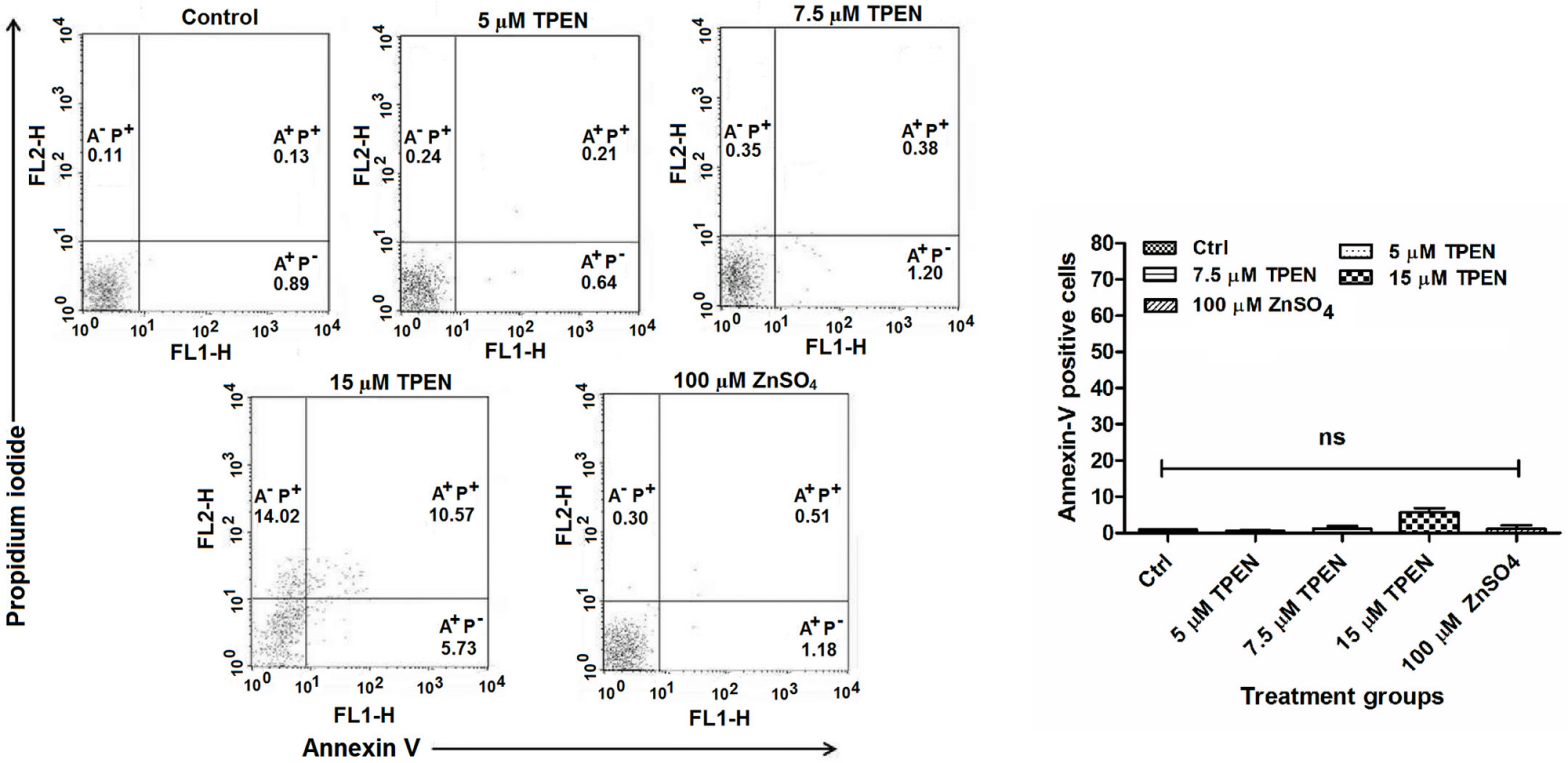
Zinc depletion failed to induce externalization of phosphatidylserine in *Leishmania* parasite. *Leishmania* parasites were treated with TPEN and ZnSO_4_ in RPMI media for 24 hr. The cells were double-stained with Annexin V-FITC and PI followed by analysis by flow cytometry as described in “Materials and methods”. Dual parametric dot plots of the Annexin-V/PI assay in different conditions as mentioned were shown in figures. The graphs were plotted and percent annexin V positive Leishmanial cells were represented. The data represents mean±SD of triplicate determinations and are representative of three independent experiments.* P<0.05, ** P<0.001, ns Not significant; as compared in zinc depleted and zinc supplemented parasites with untreated control parasites by Kruskal-Wallis test with Dunn’s multiple comparison test.

Phosphatidylserine (PS) is a phospholipid that is restricted to the inner plasma membrane leaflet under normal condition and translocated to outer membrane leaflet during apoptosis. The externalization of PS is detected by using a Ca^2+^-dependent phospholipid binding protein Annexin-V that preferentially binds PS [36, 37]. To detect externalization of PS we used Annexin V-FITC and PI staining was used for plasma membrane integrity analysis thus differentiating between apoptotic and necrotic cells. No significant differences in Annexin V-FITC^+^ and PI^+^ cells were observed in Zn-depleted, zinc supplemented and untreated control parasites after 24 hr treatment (Fig 9).

### Intracellular Zn depletion causes mitochondrial dysfunction induced apoptosis like programmed cell death

**Fig 10:**
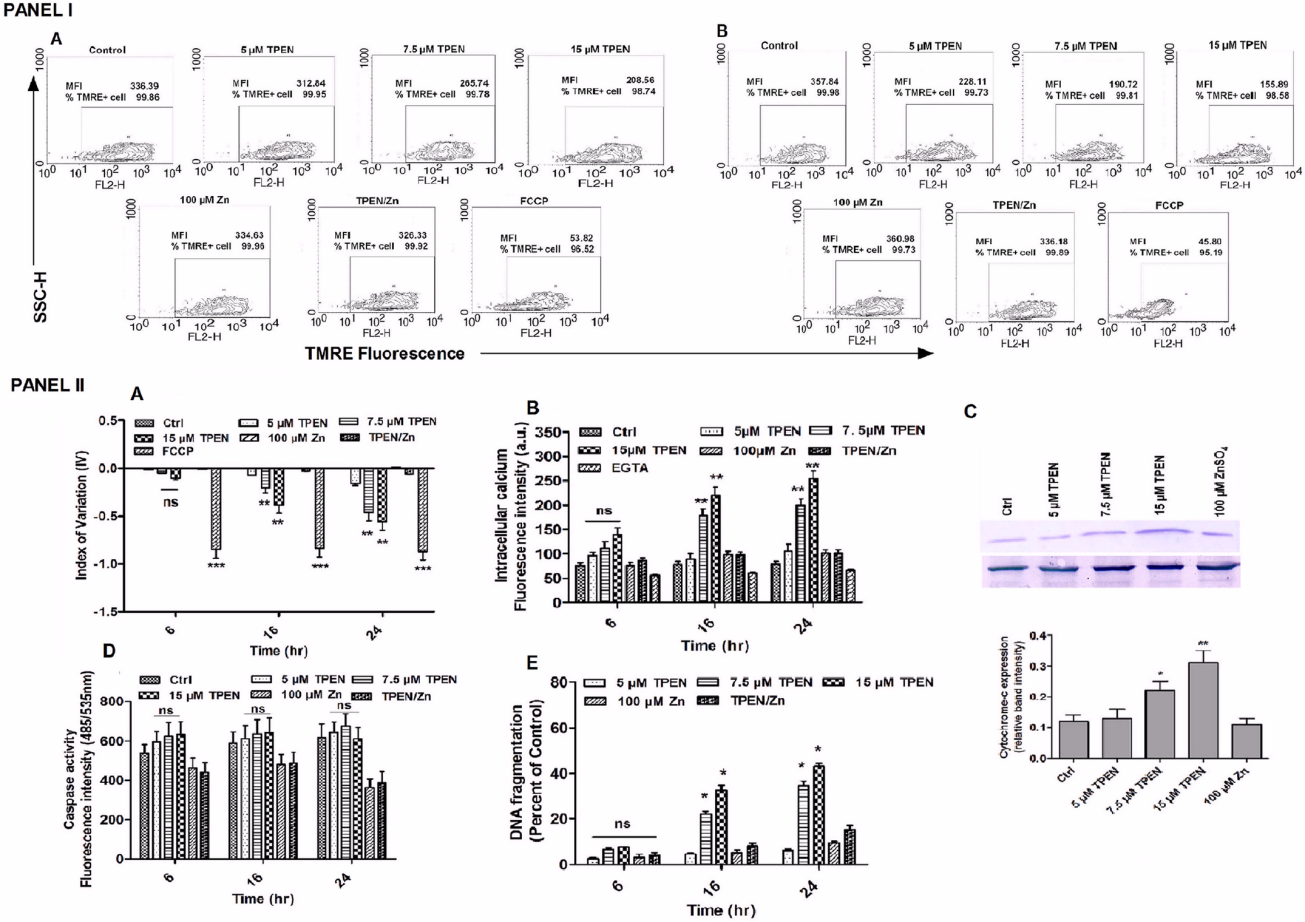
Intracellular zinc depletion causes mitochondrial dysfunction in *L*. *donovani* promastigote. **(PANEL I)** Zinc depleted and zinc supplemented *L*. *donovani* was cultivated in RPMI medium for indicated time periods and changes in mitochondrial membrane potential was determined by flow cytometry using TMRE dye. Plots are showing mean fluorescence intensity of TMRE positive cells after 16 hr (A) and 24 hr (B). FCCP was used as positive control for depolarization of mitochondrial membrane potential. **(PANEL II)** Zinc depleted and zinc supplemented *L*. *donovani* was cultivated in RPMI medium for indicated time periods and mitochondrial membrane depolarization was estimated by index of variation (IV) = (MT—MC)/MC, where MT corresponds to the median TMRE fluorescence of treated parasites and MC corresponds to the median TMRE fluorescence of control parasites (A). Intracellular calcium was measured by spectrofluorometry using Fura-2AM dye (B) and level of cytochrome c was checked in cytosolic fractions by western blotting (C) as described in “Materials & methods” section. EGTA was used as control for calcium chelation. Zinc depleted and zinc supplemented *L*. *donovani* was cultivated in RPMI medium for indicated time periods and caspase 3/7 activity was measured by spectrofluorometry as described in “Materials & methods” section. (D). DNA fragmentation was estimated by Cell death detection ELISA as described in materials and methods (E). The data represents Mean±S.D of three independent experiments in triplicates. * P< 0.05, **P< 0.001, *** P< 0.0001, ns Not significant; as compared in zinc depleted and zinc supplemented parasites with untreated control parasites by Kruskal-Wallis test with Dunn’s multiple comparison test.

As we observed that intracellular Zn^2+^ chelation by TPEN induced morphological changes and reduced cell viability in *L donovani* promastigotes we wanted to investigate the mechanism of TPEN induced cell death. Therefore we investigated mitochondrial dependent apoptotic pathway. Mitochondrial dysfunction and apoptosis are closely associated in trypanosomatids. Dysfunction of mitochondria is often associated with changes in its membrane potential. It is a key indicator of mitochondrial function that maybe either a consequence of or an early requirement for apoptosis [38, 39]. Depolarization of mitochondrial membrane potential (Δψm) is a significant characteristic of apoptosis in *L*. *donovani* [40]. Tetramethylrhodamine ethylester (TMRE) is a cell permeable fluorescent cationic dye that accumulates in healthy cells. We observed a decrease in the fluorescence intensity of TMRE in dose- and time- dependent manner in Zn depleted parasites whereas no significant changes in fluorescence was observed in parasites treated with Zn or chelator in the presence of Zn ions **(**Fig 10 **PANEL I A & B)**. Treatment with 5, 7.5 and 15 μM TPEN induced 7, 21 and 38% after 16 hr and 16, 46 & 56% depolarization in Δψm after 24 hr as indicated by their respective index of variation (P<0.001) **(**Fig 10 **PANEL II A)**. Simultaneously, when parasites were treated with FCCP fluorescence intensity was drastically decreased (P<0.0001). This result clearly demonstrated that ROS generated due to Zn chelation leads to alterations in mitochondrial membrane potential in *L*. *donovani* similar to the other studies [25, 41, 42].

Oxidative stress induces increase in cytosolic calcium level which causes mitochondrial membrane depolarization [40]. Treatment of parasites with zinc chelator gradually increased the cytosolic Ca^2+^ in dose- and time- dependent manner as observed by increase in fluorescence intensity of FURA-2AM dye whereas no significant change was observed in zinc supplemented *L*. *donovani* parasites for 0–16 hr. Here, we found ~ 2.1 fold, ~ 2.5 fold and ~ 3.2 fold increase in intracellular calcium at TPEN concentration of 5 μM, 7.5 μM and 15 μM respectively after 16 hr incubation **(**Fig 10 **PANEL II B)**. Furthermore, treatment of parasites with TPEN/Zn and Ca^2+^ chelator, EGTA decreased the intracellular Ca^2+^ to normal level. Therefore, the above findings indicate that ROS generation due to intracellular zinc depletion caused irreparable damage to calcium channels. This damage resulted into the increase in intracellular calcium followed by loss of mitochondrial membrane potential leading to an apoptosis like programmed cell death in *L*. *donovani* parasite.

By apoptotic stimuli cytochrome c, which is localized in the inner mitochondrial membrane space, is released into the cytosol after disruption of the outer mitochondrial membrane. Release of cytochrome-c was measured in cytosolic protein fraction from the parasites treated in the presence or absence of TPEN and ZnSO_4_ for 16 hr using anti-cytochrome c antibody by western blotting **(**Fig 10 **PANEL II C).** Densitometry analysis of the immunoblot showed that cytosolic cytochrome-c was ~ 2.5-fold and ~ 2.1-fold higher in the TPEN (7.5μM and 15μM) treated parasites than in Zn-supplemented or untreated control cells. From the above result it may be concluded that intracellular Zn chelation by TPEN treatment induced the ROS-mediated loss in mitochondrial membrane potential leading to increase in cytosolic Ca^2+^ and release of cytochrome c from the mitochondria into the cytoplasm.

Activation of protease and nuclease follow the disruption of Δψm responsible for dismantling of cells [38]. Therefore we investigated the change in caspase-like protease activity due to alterations in Zn level in *L*. *donovani* parasites. However, we did not find any significant activation of caspase-like activity in TPEN and ZnSO_4_ treated parasites as compared to untreated control parasites at any time and dose investigated **(**Fig 10 **PANEL II D)**.

To investigate whether intracellular Zn depletion causes DNA fragmentation in *L*. *donovani* cells, DNA fragmentation assay determining mono- and oligonucleosomes in the cytoplasmic fraction was carried out as described in materials and methods. ~ 2.2-fold and ~ 3.4-fold increase in DNA fragmentation was observed in 7.5 μM and 15 μM TPEN treated cells compared to untreated control parasites at 16 hr and ~ 3.4-fold and ~ 4.4-fold at 24 hr incubation respectively **(**Fig 10 **PANEL II E)**. Furthermore, no significant change in DNA fragmentation was observed in Zn-supplemented and TPEN/Zn-treated parasites compared to untreated control cells.

## Discussion

Zinc, being a critical co-factor of many cellular proteins, has a diverse physiological role including regulation of cell proliferation and cell death [43]. Cellular zinc homeostasis is tightly controlled and any disruptions threaten cell survival and impair cellular functions. To support essential functions cells tightly control zinc transport into and out of the cell and their distribution within cellular compartments [43, 44]. Cell death has been shown to be induced in many cell types by depleting zinc through culture in zinc-free medium or zinc chelation [15, 45–47].

Our present study demonstrated that the growth and survival of the *Leishmania* parasite is inhibited by the depletion of intracellular Zn. Whereas Zn supplementation alone or along with chelator had no inhibitory effect on survival and proliferation, indicating that the growth and proliferation of the *Leishmania* promastigotes depends on the availability of Zn. The plausible explanation for the decreased proliferation and viability by zinc chelation is due to the presence of Zn as a structural element in the enzymes involved in DNA synthesis, transcription, aminoacyl-tRNA synthesis and ribosomal functions [12]. Further to affirm that the TPEN-induced cell death was a consequence of TPEN-induced depletion of intracellular Zn not due to non-specific toxicity by TPEN or other divalent metal ions *Leishmania* cells were treated with TPEN in the presence of Zn^2+^, Cu^2+^, Ca^2+^ and Mg^2+^. Supplementation of parasites with other cations like Ca^2+^, Mg^2+^ alone could not restore the growth of parasites however presence of Cu^2+^ in the medium partially reduces the toxicity caused by Zn chelation and resulted into the partial restoration of growth and survival of the parasites. Therefore, it indicates that Zn is essential for the growth of *L*. *donovani* parasite. Findings of our experiments are in agreement with the previous reports in *Trypanosoma cruzi* [48], *L*. *braziliensis* [23] and *L*. *mexicana amazonensis* [49] which showed that the treatment of these parasites with 1,10-phenanthroline, another Zn chelating compound and metallopeptidase inhibitor reduces the growth and proliferation.

To investigate the effect of TPEN on intracellular labile Zn^2+^ we had determined intracellular Zn^2+^ level by Fluozin-3AM staining that specifically stains labile Zn pool. We observed a dose- and time-dependent flux in labile Zn^2+^ with a rapid decrease in intracellular labile Zn in first few minutes and subsequently increased after TPEN treatment. This flux in intracellular Zn may occur by increased uptake of Zn probably by upregulating the expression of Zn transporter. Recently an upregulation of Zn importer LiZIP3 in the presence of EDTA and TPEN and down regulation in the presence of Zn^2+^ has been reported in *L*. *infantum* [18]. This increase in intracellular labile Zn suggested the presence of important mechanism in *L*. *donovani* to cope up with Zn stress induced by intracellular Zn deprivation. However, this rise may also be attributed to the change in the redox state of cell as proposed by Zalewski *et al*, 1994 which states that in the later stages of apoptosis Zn bound to protein sulfhydryls are being released due to ROS generation [50]. Interestingly with increasing time the Zn level was further declined which may resulted into the reduced viability of parasites due to intracellular Zn-deficiency. TPEN has been reported to have a very high affinity for Zn which is even greater than that of endogenous zinc binding proteins such as metallothionein, and zinc finger proteins [51]. Thus the presence of TPEN, in the culture medium and in cell; blocked the attempts of parasites to maintain zinc homeostasis during the phase of declining intracellular Zn level. No such fluxes or increase in intracellular zinc was observed in Zn^2+^ supplemented parasites during this period and the parasites continued to survive and proliferate. Zinc has been suggested to be a physiological regulator of apoptosis and decline in intracellular zinc has been associated with apoptosis. Several apoptosis inducing agents have been reported to cause a decrease in LIPZ. In cells that are induced to undergo apoptosis there is a decrease in LIPZ initially however during the process of apotosis LIPZ rise. However, this change in LIPZ does not occur in necrosis [43]. Thus similar flux in LIPZ in TPEN-treated parasites but not in Zn-supplemented cells has led us to presume the involvement of apoptotic-like programme cell death (PCD) mechanisms in TPEN treated parasites as well.

ROS are considered signalling molecules which activate or regulate crucial components and regulate proteins of the PCD machinery. Thus at any moment the level of intracellular ROS can determine the fate of the cell [52]. To elucidate the reason behind reduced growth and viability, we assessed intracellular ROS generation in *L donovani* promastigotes and found that similar to LIPZ, a significant flux in intracellular ROS occurs in Zn depleted parasites compared to Zn supplemented and control cells. Although, nutrient deprivation (such as glucose, amino acid, purines and metals) has been previously shown to induce ROS generation [3], to the best of our knowledge this is the first report describing ROS generation due to Zn deprivation in *L*. *donovani*. Previously oxidative stress generation has also been shown in Zn deprived *Candida gaitii* and *Aspergillus fumigatus* [11]. Therefore reduced cell viability due to Zn deprivation can be attributed to stress induced by increased ROS generation.

Non-protein thiols, like glutathione and trypanothione; plays an important role in defense against oxidative stress caused by free radicals like ROS [3, 35]. Thus in this study, we have investigated the total intracellular thiol content which is found to be significantly reduced in Zn-depleted parasites compared to Zn-supplemented and untreated control parasites with increasing time. The decreased intracellular thiol level in Zn-depleted parasites is correlated with increased intracellular ROS production leading to decreased parasite survival. These observations prompted us to study the expression pattern of thiol metabolic pathway enzymes. Thus, we have investigated for the expression pattern of three important trypanothione synthetic enzymes namely γ-GCS, Try S and TR. We have found significantly reduced expressions of these enzymes both at transcriptional and translational level. Results of our study were similar to the study by Mandal *et al* [3] and Purkait *et al* [28] where they have also illustrated that the decreased expression of thiol metabolic pathway enzymes decreases the cell survival in *L*. *donovani* as the parasites were unable to withstand the oxidative stress and undergo ROS-dependent killing.

Zn-depletion did not increases annexin-V fluorescence intensity in treated parasites, indicating no externalization of phosphatidylserine. In contrast we found that Zn-depletion induced severe morphological alterations, LIPZ flux, ROS production and decreased intracellular thiol content. These observations may indicate apoptosis mediated cell death in *L*. *donovani* parasites. Similarly, Britta *et al* also showed that thiosemicarbazone derived from S-limonene caused apoptotic like cell death by oxidative damage and mitochondrial dysfunction in absence of phosphatidylserine exposure [36].

Intracellular zinc depletion disrupts normal functioning of mitochondria and may activate the mitochondrial/apoptosome-dependent death pathway as this is critically involved in the activity of several transcription factors and enzymes. [53–57]. Many *in vitro* studies on Zn-deficiency induced apoptosis have shown the involvement of intrinsic cell death pathway with the decrease in Δψm followed by release of cytochrome c and activation of caspase enzymes [42, 51, 52, 58–60]. The drop of the Δψm marks the point of no-return of a cell conditioned to die [52]. Consistent with the above reports, we also found that chelation of intracellular labile Zn pools leads to the activation of the apoptotic cascade in *Leishmania* promastigotes that is accompanied by the loss of mitochondrial membrane potential, increase in intracellular calcium and release of cytochrome c into cytosol. However, activation of caspase was not observed. Interestingly, these apoptosis related phenomena were not observed in Zn-supplemented parasites. Reduction in formazan formation in MTT assay serving as a measure of decreased cell viability due to reduced mitochondrial dehydrogenase activity itself indicates mitochondrial dysfunction. It is associated with a decrease in mitochondrial membrane potential, corroborated by the decreased fluorescence intensity of TPEN-treated promastigotes following TMRE staining. Further to confirm apoptosis like cell death in *L*. *donovani* we investigated DNA fragmentation analysis by cell death detection ELISA. We found significant increase in DNA fragmentation in Zn-depleted parasites compared to Zn-supplemented and untreated control parasites. Thus our observations, demonstrate that TPEN mediated Zn chelation causes apoptosis like programmed cell death in *L*. *donovani*, however sharing only some not all the classical features of eukaryotic apoptosis as seen by absence of phosphatidylserine exposure and caspase activation.

The leishmanolysin or gp63, a 60–66 kDa Zn-dependent metalloprotease abundantly expressed on the metacyclic promastigote surface; is involved in several steps of parasite-host interaction and virulence, including a protective role against the parasite degradation within macrophage phagolysosomes [22, 49]. When we investigated the effect of Zn on the parasite-macrophage interaction we found reduced interaction between THP-1-derived macrophages and Zn-depleted parasites compared to Zn-supplemented parasites as indicated by their respective association index. Similar studies in *L*. *braziliensis* and *T*. *brucei* [61] had also demonstrated decreased association index and subsequent reduced interaction and infection between these parasites and host macrophages when pre-treated with PHEN. Inhibition of gp63 proteolytic activity substantiated the mechanism of reduced interaction between trypanosomatid parasite and host macrophage [22]. In our study the reduced interaction between promastigote and macrophage may also be attributed to Zn-chelation induced inhibition of Zn-metalloprotease gp63 proteolytic activity that is required for successful interaction and subsequent infection.

Thus, our study showed the importance of availability of Zn for the survival and proliferation of *L*. *donovani*. It emphasized about the susceptibility of *Leishmania* promastigotes to intracellular Zn deprivation and disturbance in the intracellular Zn homeostasis results into the death of *L*. *donovani* parasite. *Leishmania* growth depends on a constant intake of Zn and leishmanial Zn depletion increases the synthesis of harmful reactive oxygen species.

From the findings of this study it can be concluded that intracellular zinc homeostasis has crucial role in the growth, survival and virulence of *Leishmania* parasites. Disruption in zinc homeostasis results into apoptosis-like cell death. Our study is the first report elucidating the role of intracellular zinc in the cell survival and apoptosis in *L*. *donovani* promastigotes. The molecular mechanism for Zn regulated apoptosis-like cell death involves ROS-mediated caspase independent mitochondrial dysfunction through influx of intracellular calcium leading to depolarization of mitochondrial membrane potential with cytosolic cytochrome c release and subsequent DNA degradation. Thus, regulation of Zn homeostasis and Zn acquisition can be explored as new therapeutic targets for the development of new generation antileishmanial drugs.

## References

[1] Alvar J, Vélez ID, Bern C, Herrero M, Desjeux P, Cano J, et al Leishmaniasis worldwide and global estimates of its incidence. PLoS One. 2012; 7: e35671 10.1371/journal.pone.0035671 22693548 PMC3365071

[2] WHO, Control of the Leishmaniasis. World Health Organ Tech Reser. 2010; (949): p. xii–xiii, back cover. 21485694

[3] Mandal A, Das S, Roy S, Ghosh AK, Sardar AH, Verma S, et al Deprivation of L-Arginine Induces Oxidative Stress Mediated Apoptosis in *Leishmania donovani* Promastigotes: Contribution of the Polyamine Pathway. PLoS Negl Trop Dis. 2016; 10:1 e0004373 10.1371/journal.pntd.0004373 26808657 PMC4726550

[4] Desjardins M, Descoteaux A. Survival strategies of *Leishmania donovani* in mammalian host macrophages. Res. Immunol. 1998; 149: 689–692. 9851525 10.1016/s0923-2494(99)80040-6

[5] Kehl-Fie TE, Skaar EP. Nutritional immunity beyond iron: a role for manganese and Zn. Curr Opin Chem Biol. 2010 4; 14(2): 218–224. 10.1016/j.cbpa.2009.11.008 20015678 PMC2847644

[6] Sandstead HH. Zinc is essential for brain development and function. The Journal of Trace Elements in Experimental Medicine. 2003; 16:165‐173.

[7] Stefanidou M, Maravelias C, Dona A, Spiliopoulou C. Zinc: a multipurpose trace element. Archives of Toxicology. 2006; 80: 1–9. 10.1007/s00204-005-0009-5 16187101

[8] Prasad AS, Oberleas D. Thymidine kinase activity and incorporation of thymidine into DNA in zinc-deficient tissue. Journal of Laboratory and Clinical Medicine. 1974; 81:634–639. 4817783

[9] Outten CE, O’Halloran TV. Femtomolar sensitivity of metalloregulatory proteins controlling Zn homeostasis. Science. 2001; 292:2488–2492. 10.1126/science.1060331 11397910

[10] Andreini C, Bertini I, Cavallaro G, Holliday GL, Thornton JM. Metal ions in biological catalysis: from enzyme databases to general principles. J. Biol. Inorg. Chem. 2008; 13:1205–1218. 10.1007/s00775-008-0404-5 18604568

[11] Vicentefranqueira R, Amich J, Laskaris P, Ibrahim-Granet O, Latgé JP, Toledo H, et al Targeting Zn homeostasis to combat *Aspergillus fumigatus* infections. Frontiers of Microbiology. 2015; 6:160, 1–7. 10.3389/fmicb.2015.00160 PMC4343018 25774155

[12] Beyersmann D, Haase H. Functions of zinc in signaling, proliferation and differentiation of mammalian cells. BioMetals. 2001; 14: 331–341. 11831463 10.1023/a:1012905406548

[13] Wu BW. N,N,N’,N’-tetrakis (2-pyridylmethyl) ethylenediamine-induced depletion of the labile intracellular pool of zinc suppressed the growth of MDA-MB-231 human breast cancer cells. 2003. UBC (Thesis).

[14] Coyle P, Zalewski PD, Philcox JC. Measurement of zinc in hepatocytes by using a flurosence probe, zinquin: relationship to metallothionein and intracellular zinc. Biochemical Journal. 1994; 303: 781–786. 7980447 10.1042/bj3030781 PMC1137615

[15] Zalewski PD, Forbes IJ, Betts WH. Correlation of apoptosis with change in intracellular labile Zn(II) using zinquin [(2-methyl-8-p-toluene-sulphonamido-6-quinolyloxy)acetic acid], a new specific fluorescent probe for Zn(II). Biochemical Journal. 1993; 296(Pt2): 403–408. 8257431 10.1042/bj2960403 PMC1137710

[16] Paski SC, Xu Z. Labile intracellular zinc is associated with 3T3 cell growth. Journal of Nutritional Biochemical. 2001; 12: 655–661. 10.1016/s0955-2863(01)00188-7 12031259

[17] Olivier M, Atayde VD, Isnard A, Hassani K, Shio MT. *Leishmania* virulence factors: focus on the metalloprotease GP63. Microbes Infect. 2012; 14: 1377–1389. 10.1016/j.micinf.2012.05.014 22683718

[18] Carvalho S, Barreira da Silva R, Shawki A, Castro H, Lamy M, Eide D, et al LiZIP3 is a cellular Zn transporter that mediates the tightly regulated import of Zn in *Leishmania infantum* parasites Molecular Microbiology. 2015; 10.1111/mmi.12957 25644708 PMC4964273

[19] Silva MS, Barata L, Ferreira AI, Roma S, Tomas AM, Freira AP et al Catalysis and structural properties of *Leishmania infantum* glyoxalase II: trypanothionne specificity and phylogeny. Biochemistry. 2008; 47(1): 195–204. 10.1021/bi700989m 18052346

[20] Trincao J, Silva MS, Barata L, Bonifacio C, Carvalho S, Tomas AM, et al Purification, crystallization and preliminary X-ray diffraction analysis of the glyoxalase II from *Leishmania infantum*. Acta Crystallogr Sect F Struct Biol Cryst Commun. 2005; 62(8): 805–7. 10.1107/S1744309106027539 16880563 PMC2242913

[21] Bafghi AF, Noorbala M, Noorbala MT, Aghabagheri M. Anti-Leishmanial Effect of Zn Sulphate on the Viability of *Leishmania tropica* and *L*. *major* Promastigotes. Jundishapur J Microbiol. 2014; 7(9): e11192 10.5812/jjm.11192 25485055 PMC4255370

[22] Santos ALS, Sodré CL, Valle RS, Silva BA, Abi-Chacra ÉA, Silva LV, et al Antimicrobial Action of Chelating Agents: Repercussions on the Microorganism Development, Virulence and Pathogenesis. Current Medicinal Chemistry. 2012; 19(1). 10.2174/092986712800609788 22455582

[23] Lima AKC, Elias CGR, Souza JEO, Santos ALS, Dutra PML. Dissimilar peptidase production by avirulent and virulent promastigotes of *Leishmania braziliensis*: inference on the parasite proliferation and interaction with macrophages. Parasitology. 2009; 136:1179–1191 10.1017/S0031182009990540 19631015

[24] Mosmann T. Rapid colorimetric assay for cellular growth and survival: application to proliferation and cytotoxicity assays. J Immunol Methods. 1983; 65: 55–63. 6606682 10.1016/0022-1759(83)90303-4

[25] Mesquita-Rodrigues C, Menna-Barreto RFS, Sabo´ ia-Vahia L, Da-Silva SAG, de Souza EM, et al Cellular Growth and Mitochondrial Ultrastructure of *Leishmania (Viannia) braziliensis* Promastigotes Are Affected by the Iron Chelator 2,2-Dipyridyl. PLoS Negl Trop Dis. 2013; 7(10): e2481 10.1371/journal.pntd.0002481 24147167 PMC3798463

[26] Haase H, Ober-Blo¨baum JL, Engelhardt G, Hebel S, Heit A, Heine H, Rink L. Zinc Signals Are Essential for Lipopolysaccharide-Induced Signal Transduction in Monocytes. The Journal of Immunology. 2008; 181: 6491–6502. 18941240 10.4049/jimmunol.181.9.6491

[27] Mukhopadhyay R, Dey S, Xu N, Gage D, Lightbody J, Ouellette M, and Rosen BP. Trypanothione overproduction and resistance to antimonials and arsenicals in *Leishmania*. Proc. Natl. Acad. Sci. 1996; 93:10383–10387 8816809 10.1073/pnas.93.19.10383 PMC38393

[28] Purkait B, Kumar A, Nandi N, Sardar AH, Das S, Kumar S et al Mechanism of amphotericin B resistance in clinical isolates of *Leishmania donovani*. Antimicrob Agents Chemother. 2012; 56: 1031–41. 10.1128/AAC.00030-11 22123699 PMC3264217

[29] Sardar AH, Das S, Agnihorti S, Kumar M, Ghosh AK, Abhishek K et al Spinigerin induces apoptotic like cell death in a caspase independent manner in *Leishmania donovani*. Exp Parasitol. 2013; 135:715–25. 10.1016/j.exppara.2013.10.011 . 24184774

[30] Gannavaram S, Vedvyas C, Debrabant A. Conservation of the pro-apoptotic nuclease activity of endonuclease G in unicellular trypanosomatid parasites. J Cell Sci. 2008; 121: 99–109. 10.1242/jcs.014050 18073240

[31] Equbal A, Suman SS, Anwar S, Singh KP, Zaidi A, et al (2014) Stage-Dependent Expression and Up-Regulation of Trypanothione Synthetase in Amphotericin B Resistant *Leishmania donovani*. PLoS ONE 9(6): e97600 10.1371/journal.pone.0097600 24901644 PMC4046939

[32] Chimienti F, Aouffen M, Favier A, Seve M. Zinc Homeostasis-regulating Proteins: New Drug Targets for Triggering Cell Fate. Current Drug Targets. 2003; 4(4):323–328 12699353 10.2174/1389450033491082

[33] Li YM, Shi J, Wu X, Luo ZF, Wang FL, Guo QX. Tracing of intracellular zinc (II) fluorescence flux to monitor cell apoptosis by using FluoZin-3AM. Cell Biochem Funct. 2009; 27: 417–423. 10.1002/cbf.1598 19784961

[34] Patricia I. Oteiza. Zinc and the modulation of redox homeostasis. Free Radic Biol Med. 2012; 53 (9):1748–1759. 10.1016/j.freeradbiomed.2012.08.568 22960578 PMC3506432

[35] Mandal G, Wyllie S, Singh N, Sundar S, Fairlamb AH, Chatterjee M. Increased levels of thiols protect antimony unresponsive Leishmania donovani field isolates against reactive oxygen species generated by trivalent antimony. Parasitology. 2007;134: 1679–87. 10.1017/S0031182007003150 17612420 PMC3409873

[36] Britta EA, Scariot DB, Falzirolli H, Ueda-Nakamura T, Conceição Silva C, Dias Filho BP, Borsali R, Nakamura CV. Cell death and ultrastructural alterations in *Leishmania amazonensis* caused by new compound 4-Nitrobenzaldehyde thiosemicarbazone derived from S-limonene. BMC Microbiology 2014, 14(236). 10.1186/s12866-014-0236-0 25253283 PMC4188478

[37] Islamuddin M, Chouhan G, Tyagi M, Abdin MZ, Sahal D, Afrin F. Leishmanicidal activities of *Artemisia annua* leaf essential oil against Visceral Leishmaniasis. Front. Microbiol. 2014; 5(626). 10.3389/fmicb.2014.00626 25505453 PMC4243575

[38] Smirlis D, Duszenko M, Ruiz AJ, Scoulica E, Bastien P, Fasel N, Soteriadou K. Targeting essential pathways in trypanosomatids gives insights into protozoan mechanisms of cell death. Parasites & Vectors. 2010; 3(107). 10.1186/1756-3305-3-107 PMC3136144 21083891

[39] Foucher AL, Rachidi N, Gharbi S, Blisnick T, Bastin P, Pemberton IK, et al Apoptotic Marker Expression in the Absence of Cell Death in Staurosporine-Treated *Leishmania donovani*. Antimicrobial Agents and Chemotherapy. 2013; 57(3). 10.1128/AAC.01983-12 PMC3591890 23263009

[40] Mukherjee SB, Das M, Sudhandiran G, Shaha C. Increase in cytosolic Ca2+ levels through the activation of non-selective cation channels induced by oxidative stress causes mitochondrial depolarization leading to apoptosis-like death in *Leishmania donovani* promastigotes. J Biol Chem. 2002; 277: 24717–24727. 10.1074/jbc.M201961200 11983701

[41] Mehta A, Shaha C. Apoptotic death in *Leishmania donovani* promastigotes in response to respiratorychain inhibition: complex II inhibition results in increased pentamidine cytotoxicity. J Biol Chem. 2004; 279: 11798–813. 10.1074/jbc.M309341200 14679210

[42] Hashemi M, Ghavami S, Eshraghi M, Booy EP, Los M, Hashemi M, et al Cytotoxic effects of intra and extracellular zinc chelation on human breast cancer cells. European Journal of Pharmacology. 2007; 557: 9–19. 10.1016/j.ejphar.2006.11.010 17169355

[43] Truong-Tran AQ, Ho LH, Chai F, Zalewski PD. Cellular Zn Fluxes and the Regulation of Apoptosis/Gene-Directed Cell Death. J. Nutr. 2000; 130: 1459S—1466S 10801960 10.1093/jn/130.5.1459S

[44] Maret W. Molecular aspects of human cellular zinc homeostasis: redox control of zinc potentials and zinc signals. Biometals. 2009; 22:149–157. 10.1007/s10534-008-9186-z 19130267

[45] Martin SJ, Mazdai G, Strain JJ, Cotter TG, Hannigan BM. Programmed cell death (apoptosis) in lymphoid and myeloid cell lines during zinc deficiency. Clin Exp Immunol. 1991; 83:338–343. 1993365 10.1111/j.1365-2249.1991.tb05639.x PMC1535271

[46] Treves S, Trentini PL, Ascanelli M, Bucci G, Di Virgilio F. Apoptosis is dependent on intracellular zinc and independent of intracellular calcium in lymphocytes. Exp Cell Res. 1994; 211:339–343. 10.1006/excr.1994.1096 8143781

[47] Devergnas S, Chimienti F, Naud N, Pennequin A, Coquerel Y, Chantegrel J, Favier A, Seve M. Differential regulation of zinc efflux transporters ZnT-1, ZnT-5 and ZnT-7 gene expression by zinc levels: a real-time RT-PCR study. Biochem Pharmacol. 2004; 68:699–709. 10.1016/j.bcp.2004.05.024 15276077

[48] Cuevas IC, Cazzulo JJ, Sánchez DO. GP63 homologues in *Trypanosoma cruzi*: surface antigens with metalloprotease activity and a possible role in host cell infection. Infect Immun, 2003; 71: 5739–5749. 10.1128/IAI.71.10.5739-5749.2003 14500495 PMC201075

[49] Seay MB, Heard PL, Chaudhuri G. Surface Zn-proteinase as a molecule for defense of *Leishmania mexicana amazonensis* promastigotes against cytolysis inside macrophage phagolysosomes. Infect. Immun. 1996; 64:5129–5137. 8945556 10.1128/iai.64.12.5129-5137.1996 PMC174498

[50] Zalewski PD, Forbes IJ, Seamark RF, Borlinghaus R, Betts WH, Lincoln SF, Ward AD. Flux of intracellular labile zinc during apoptosis (gene-directed cell death) revealed by a specific chemical probe, Zinquin. Chem. Biol. 1994b; 153–161. 9383385 10.1016/1074-5521(94)90005-1

[51] Clegg MS, Hanna LA, Niles BJ, Momma TY, Keen CL. Zinc-deficiency-induced cell death. IUBMB Life. 2005; 57(10): 661–669. 10.1080/15216540500264554 16223705

[52] Mignotte B, Vayssiere JL. Mitochondria and apoptosis. Eur. J. Biochem. 1998; 252: 1–15. 9523706 10.1046/j.1432-1327.1998.2520001.x

[53] Brouckaert G, Kalai M, Saelens X, Vandenabeele P. Apoptotic pathways and their regulation In: Los M., Gibson S.B. (Eds.), Apoptotic Pathways as Target for Novel Therapies in Cancer and Other Diseases. 2005; Springer Academic Press, New York.

[54] Ghavami S, Kerkhoff C, Los M, Hashemi M, Sorg C, Karami-Tehrani F. Mechanism of apoptosis induced by S100A8/A9 in colon cancer cell lines: the role of ROS and the effect of metal ions. J. Leukoc. Biol. 2004; 76:169–175. 10.1189/jlb.0903435 15075348

[55] Ghavami S, Hashemi M, Kadkhoda K, Alavian SM, Bay GH, Los M. Apoptosis in liver diseases—detection and therapeutic applications. Med. Sci. Monit. 2005b; 11: RA337–RA345. 16258409

[56] Krzemieniecki K, Szpyt E, Rashedi I, Gawron K, Los M. Targeting of solid tumors and blood malignancies by antibody-based therapies—EGFR-pathway as an example. Centr. Eur. J. Biol. 2006; 1:167–182.

[57] Sensi SLS, Ton0That D, Sullivan PG, Jonas EA, Gee KR, Kaczmarek LK, Weiss JH. Modulation of mitochondrial function by endogenous Zn^2+^ pools. PNAS. 2003; 100 (10): 6157–6162. 10.1073/pnas.1031598100 12724524 PMC156342

[58] Duffy JY, Miller CM, Rutschilling GL, Ridder GM, Clegg MS, Keen CL, Daston GP. A decrease in intracellular zinc level precedes the detection of early indicators of apoptosis in HL-60 cells. Apoptosis. 2001; 6: 161–172. 11388665 10.1023/a:1011380508902

[59] Barczyk K, Kreuter M, Pryjma J, Booy EP, Maddika S, Ghavami S, Berdel WE, Roth J, Los M. Serum cytochrome c indicates in vivo-apoptosis and it can serve as a prognostic marker during cancer therapy. Int. J. Cancer. 2005; 114:167–173. 10.1002/ijc.21037 15800951

[60] Maddika S, Booy EP, Johar D, Gibson SB, Ghavami S, Los M. Cancer-specific toxicity of apoptin is independent of death receptors but involves the loss of mitochondrial membrane potential and the release of mitochondrial cell death mediators by a Nurr77-dependent pathway. J Cell Sci. 2005; 118: 4485–4493. 10.1242/jcs.02580 16179607

[61] Bangs JD, Ransom DA, Nimick M, Christie G, Hooper NM. *In vitro* cytocidal effects on *Trypanosoma brucei* and inhibition of *Leishmania major* GP63 by peptidomimetic metalloprotease inhibitors. Mol. Biochem. Parasitol. 2001; 114:111–117. 11356520 10.1016/s0166-6851(01)00244-4

